# Extracting Summary Statistics of Rapid Numerical Sequences

**DOI:** 10.3389/fpsyg.2021.693575

**Published:** 2021-10-01

**Authors:** David Rosenbaum, Moshe Glickman, Marius Usher

**Affiliations:** ^1^School of Psychological Sciences, Tel-Aviv University, Tel Aviv, Israel; ^2^Department of Experimental Psychology, University College London, London, United Kingdom; ^3^Max Planck Centre for Computational Psychiatry and Ageing Research, London, United Kingdom

**Keywords:** numerical cognition, computational modeling, decision making, averaging, population coding, summary statistics

## Abstract

We examine the ability of observers to extract summary statistics (such as the mean and the relative-variance) from rapid numerical sequences of two digit numbers presented at a rate of 4/s. In four experiments (total *N* = 100), we find that the participants show a remarkable ability to extract such summary statistics and that their precision in the estimation of the sequence-mean improves with the sequence-length (subject to individual differences). Using model selection for individual participants we find that, when only the sequence-average is estimated, most participants rely on a holistic process of frequency based estimation with a minority who rely on a (rule-based and capacity limited) mid-range strategy. When both the sequence-average and the relative variance are estimated, about half of the participants rely on these two strategies. Importantly, the holistic strategy appears more efficient in terms of its precision. We discuss implications for the domains of two pathways numerical processing and decision-making.

## Introduction

Imagine a stock-market operator viewing a rapid sequence of stock returns on which she needs to make a fast buy/sell decision, or alternatively, a person who faces a crowd of people, each exhibiting a distinct emotional expression toward the person, who then needs to decide if to approach or not. In situations such as this, the rapid extraction of summary statistics of the elements (numerical returns or emotional expressions), in particular their average, has obvious advantages. Recent research over the last two decades has convincingly demonstrated that humans have a remarkable ability to extract summary statistics from large sets of visual elements, briefly presented together or in fast sequence, with regards to visual properties such as size, orientation, and even emotional expression (Ariely, 2001; Dakin, 2001; Parkes et al., 2001; Chong and Treisman, 2005; Haberman and Whitney, 2011; Robitaille and Harris, 2011; Allik et al., 2013; Khayat and Hochstein, 2018; Rosenbaum et al., 2021). Similarly, it has been reported that humans can extract the arithmetic average (the simplest summary statistic) from rapid numerical (or numerosity) sequences (Malmi and Samson, 1983; Brezis et al., 2015; Katzin et al., 2021). For example, Brezis et al. (2018) reported that human observers are able to provide quite accurate estimates of numerical average for sequences of 2-digit numbers (sequence length 6–12) that are presented at a rate of 4/sec. Moreover, they demonstrated the precision of these estimates increases with sequence length, and decreases with the sequence mean and variance. Finally, they proposed a model based on a population of broadly number-magnitude detectors (Dehaene, 1992, 2007; Feigenson et al., 2004), which accounted for all these data patterns (see next section). Furthermore, in a recent study, Hadar et al. (2021) have supported the idea that averaging is a type of gist extraction that is facilitated by an “abstract” mind-set (Gilead et al., 2019).

While this extensive research has focused on the extraction of an ensemble-average, there is less research on the extraction of higher order summary statistics, such as the variance (Kareev et al., 2002; Morgan et al., 2008; Solomon, 2010; Bronfman et al., 2015; Ward et al., 2016), in particular for numerical sequences. This shortage stands out, given the well-known impact of the variance of a payoff-set on risk-preferences (Markowitz, 1952; Weber, 2010; Summerfield and Tsetsos, 2012; Zeigenfuse et al., 2014; Erev et al., 2017; Glickman et al., 2018; Vanunu et al., 2019, 2020). One idea that has been proposed in recent research is that summary statistics (such as the sum, the average or the variance) are automatically extracted and relied on to form intuitive preferences (Betsch et al., 2001; De Gardelle and Summerfield, 2011; Brusovansky et al., 2019; Vanunu et al., 2019). Note, however, that in order to account for risk-preferences, the intuitive system (Glöckner and Betsch, 2008) needs to extract not only the average but also a measure of the alternative-variance (Weber, 2010; Vanunu et al., 2019). Finally, intuitive decisions between complex alternatives are often subject to marked individual differences (Newell and Shanks, 2003; Glöckner and Betsch, 2008; Betsch and Glöckner, 2010; Brusovansky et al., 2018, Brusovansky et al., 2019). While some people form preferences that reflect summary statistics, others form preferences that are based on simplifying heuristics that focus on a single aspect of the information (e.g., *Take The Best*; *TTB*). Experimental context is another factor that appears to affect the weight that people give to outlier elements^1^ (De Gardelle and Summerfield, 2011; Vandormael et al., 2017; Vanunu et al., 2020). Similarly, Vanunu et al. (2019) have shown that the weight by which outlier elements contribute to risk preferences depends on how the evaluations are made, one at a time or in groups (implying a complexity cost to the estimations of several summary statistics, in parallel). It is thus important to examine if similar individual differences take place in the explicit estimation of summary statistics and to better understand the factors that determine the type of the estimation strategy a person is likely to deploy.

Motivated by these shortcomings, the aim of this paper was to probe further into the capacity of human observers to extract summary statistics of rapid numerical sequences. In particular, we extend previous investigations with regards to the following questions: (i) Can human observers extract higher order summary statistics (such as the *relative* sequence-variance^2^) at the same time as they extract the average? (ii) What types of mechanisms do people deploy when making such estimations? We contrast between holistic frequency-based estimations and sequential rule-based and working-memory limited strategies such as the *mid-range;* see next section for details. (iii) Are there individual differences in the mechanism or the strategy that human observers deploy in these tasks? (iv) Does the frequency with which participants deploy such strategies depend on the type of summary statistic that are required and on the distribution of values that are used as samples, and (v) which of those strategies (or mechanisms) result in more efficient estimations?

We start with a brief computational section that provides the motivation for our experiments. Following, we present four experiments in which human observers were asked to evaluate summary statistics of rapid (4 Hz) numerical sequences. In Experiment 1 (a and b) only the average is evaluated, while in Experiments 2 and 3, the participants are required to make two estimations for each sequence: average and relative-variance in Experiment 2, and average and confidence in Experiment 3. Finally, we present a computational analysis of the data, which is focused on individual differences with regards to the estimation mechanism and we examine their relative efficiencies.

## Computational Modeling of Numerical Averaging: Dependency on Sequence-Length

**Figure 1:**
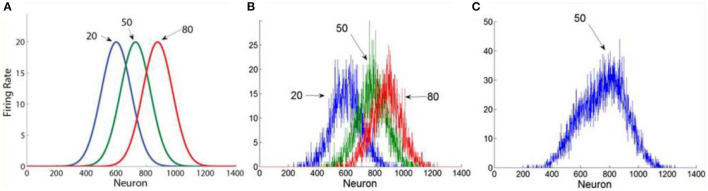
The population averaging model. **(A)** Numbers activate broadly tuned number- magnitude detectors. **(B)** In this illustration we show the noisy population profile in response to the presentation of an example sequence of 3 numbers: 20, 50, 80. **(C)** The estimation of the average is based on the central of mass of the population response (reproduced from Brezis et al., 2015).

We are focusing on the intuitive averaging of the type that people can make for rapid sequences (four per second) of two digit numbers under time pressure (see section Methods of Experiment 1). This belongs to the domain of approximate numerical estimation (Dehaene, 1992; Dehaene et al., 1998; Feigenson et al., 2004) and excludes an explicit computation of the average based on summing the numbers and division by the sequence length. Malmi and Samson (1983) considered two alternative ways of computing a sequence average: (i) a running average with decreasing weights, (ii) the estimation of the “center of mass” from a frequency distribution, and they concluded in favor of the latter. More recently, Brezis et al. (2015), Brezis et al. (2018) *population averaging* model (see Figure 1 for illustration), provides a computationally explicit mechanism that generates a noisy frequency representation of a numerical sequence and estimates its center of mass.

Accordingly, when presented with a sequence of numbers, the sequence's average is estimated from the population-averaging of the number-magnitude tuned activation profile, by weighting the contribution of each detector's activity according to its preferred number magnitude (Georgopoulos et al., 1986). At display's offset, the center of mass is estimated by summing each neuron's detector's activation multiplied by its preferred magnitude and dividing by the sum of the overall network's activity (see Brezis et al., 2018, for a biological plausible neural implementation):


(1) $$\begin{array}{l} Mean=\frac{\left(\sum F_{i}\cdot T_{i}\right)}{\sum F_{i}} \end{array}$$

where for each detector *i, F* = firing rate; *T* = the detector's preferred magnitude. The sum is taken over all numerosity detectors.

**Figure 2:**
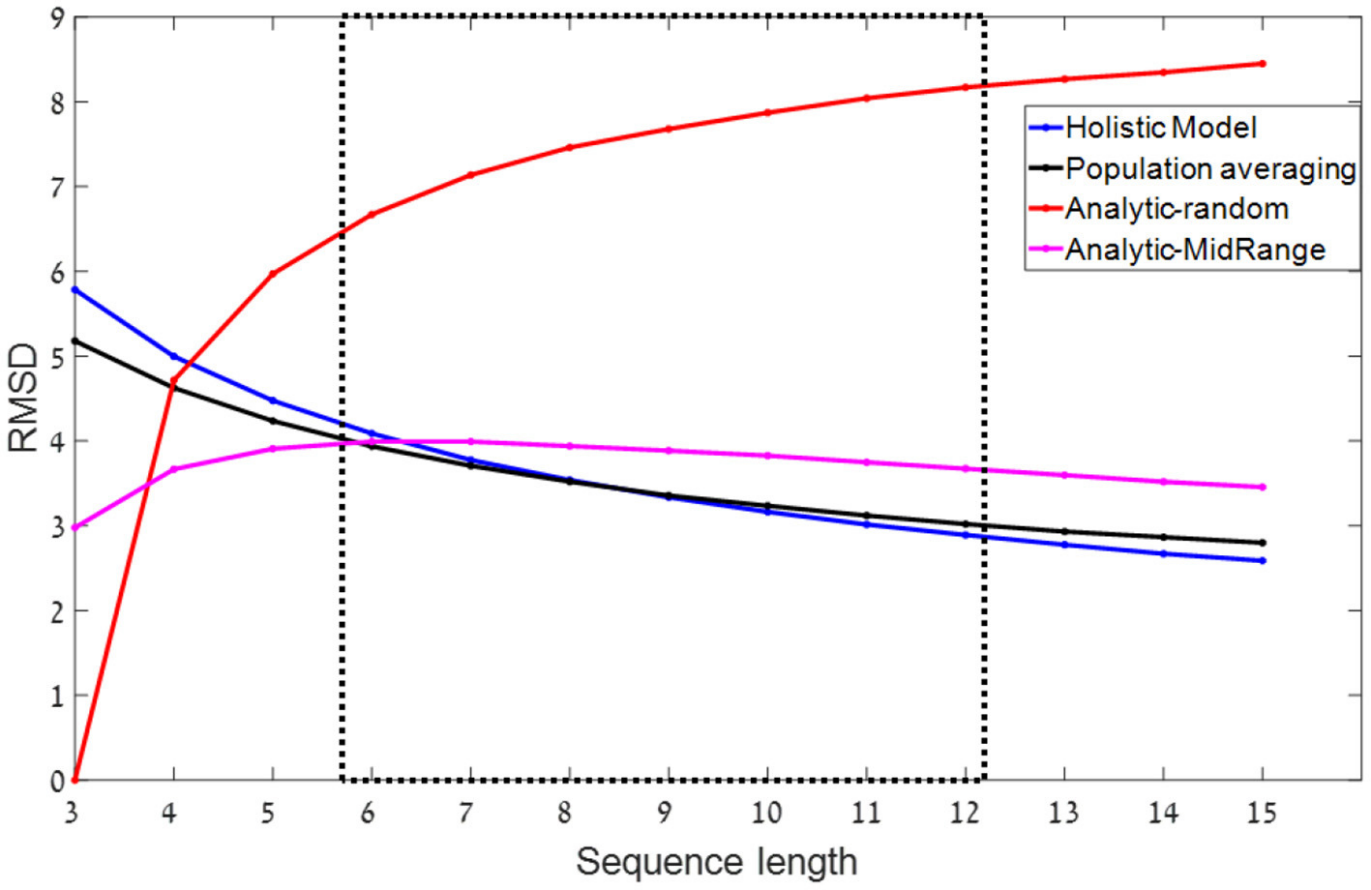
RMSD (root mean square deviations) as a function of sequence length- predictions for different models. The dashed rectangle indicates the relevant range of sequence-lengths in our experiments. In all simulations, sets of n numbers in the range of 0–100 are sampled from a uniform distribution. Blue and black: Normative-holistic and population averaging models. Both models show similar predictions in which accuracy improves as sequence length increases. Red: Analytic-random model with *k* = 3. Accuracy deteriorates as sequence length increases. Magenta: Mid-range model. Non-monotonic function. Shows a mild improvement as sequence length increases in the relevant experimental range.

The population averaging model makes a particular prediction on how the precision of the estimate depends on the length of the sequence. As the sequence-length (*n*), increases, the frequency representation becomes less noisy (due to Poisson variability of neural detectors, which decreases with the activation magnitude) and therefore the precision of the estimate increases (Figure 2). This is thus equivalent to a computation of the average, based on noisy representations of its magnitudes.


(2) $$\begin{array}{l} Mean=\frac{\sum_{i=1}^{n}X_{i}+\varepsilon_{i}}{n},\varepsilon N\left(0,\sigma^{2}\right) \end{array}$$

where, *X*_*i*_ is the *i*th item of the sequence and σ^2^is the variance of the encoding noise.

For simplification, we will here approximate the population averaging with this simplified model, which we label the *normative-holistic averaging model*. Normativity here corresponds to the property that all the items are used and weighted equally in the estimation of the average, while holism involves the idea that the estimation does not rely on a sequential application of symbolic rules and is not subject to WM capacity limitations [see contrast with heuristic models below, and Glöckner and Betsch (2008) for a similar model that is normative and yet automatic]. The *normative-holistic* model predicts that the precision of estimation would improve with $\frac{1}{\sqrt{n}}$ (Figure 2 blue line), which results from the averaging of the encoding noise, and is similar to that of the population-averaging model (Figure 2 black line).

We contrast the normative-holistic model estimation with a heuristic computation based on symbolic representations of the numbers that are subject to working memory capacity limitations (Ashcraft, 1992). The simplest such model, assumes that out of the *n*-numbers, the observers are able to remember a few random samples (2 or 3) and use them to evaluate the average of the sequence by computing their mean.^3^ For the case in which the samples stored in working memory are random, we label this the *analytical-random* model, as it relies on explicit (rule based) computation of the average based on what is available in working memory.^4^ This analytic-random model is a variant of the procedural arithmetic model (Anderson et al., 1996), in which procedural operations are serially performed on symbols available in WM. Since with increasing *n*, the few WM-samples provide a less accurate representation of the set, the precision of the analytical-random model decreases with *n* (Figure 2, red line for *k* = 3).

It is possible, however, to consider a more sophisticated analytic version of this strategy, which still computes the average based on few (e.g., two samples), but selects those samples in a non-random way (Myczek and Simons, 2008; but see Chong et al., 2008). For example, an efficient way to select two samples is to select the maximum and minimum of the sequence, resulting in the so called, *mid-range* heuristic.^5^ As shown in Figure 2 (magenta line), the mid-range heuristic predicts a milder improvement with sequence-length (for *n* > 7), since the mid-range provides a better estimate of the average, the longer the sequence is. Note that despite using one less samples (2 instead of 3) the mid-range precision is higher than that of the random-analytic with *n* > 3. However, this advantage comes with a cost. For the case that the numbers are selected from a non-symmetric, skewed distribution, the mid-range strategy predicts systematic deviations.

### Summary of Computational Contrasts Between Estimations Strategies

We have contrasted several mechanisms that observers can deploy to estimate the average of a rapid numerical sequence. The normative-holistic mechanism assumes each item contributes to the estimate but is subject to a potentially large encoding noise, which averages out with *n*. The analytical-random model assumes an exact (symbolic) computation of the average based on few (2–3) random samples, and it predicts precision to decrease with *n*. Finally, the mid-range model, predicts a milder improvement in the precision estimate with *n* (for *n* > 7), but it also predicts that only the extreme values contribute to the average estimate. Thus, to contrast between estimations strategies, we will focus on how the precision of the estimate (computed either as RMSD or as a Pearson correlation between actual and estimated sequence-average^6^) depends on sequence-length. In addition, we focus on the decision weights of the ranked items (De Gardelle and Summerfield, 2011; Vandormael et al., 2017; Vanunu et al., 2020), to probe the deployment of mid-range strategies.^7^ Finally, we wish to examine if the estimation mechanism varies with task complexity (e.g., estimating the average only or both the average and the relative-variance, Vanunu et al., 2019) or with the distribution from which the values are sampled, which is likely to modulate gains based on prior expectations (Summerfield and De Lange, 2014).

To anticipate our results, we found that observers can estimate relative-variance of rapid numerical sequences remarkably well, while they also estimate the sequence average. Moreover, while we find individual differences in the estimation strategy, most observers deploy a holistic mechanism when the task requires average estimation only, and about half of them deploy such a holistic mechanism even when they estimate both the average and the relative variance. Furthermore, we found that the likelihood of these two strategies is affected by the distribution from which the values are sampled. Finally, we find that the participants who deploy the holistic mechanism tend to be more precise in their estimations.

## Experiments

**Figure 3:**
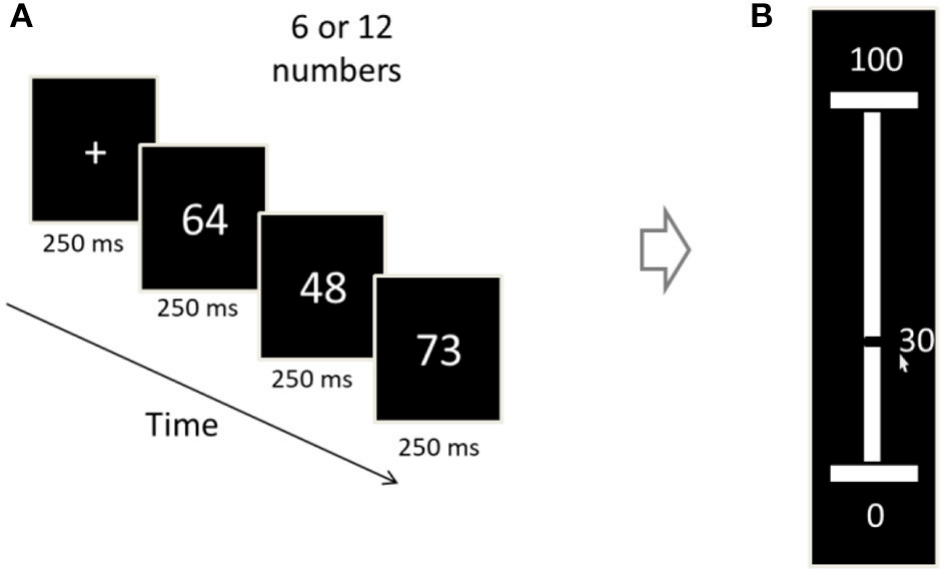
Illustration of an experimental trial (Exp. 1–2). **(A)** Each trial begins with a 250 ms fixation cross, after which a sequence of two-digit numbers is presented (250 ms/numeral). The sequence-length is 6 or 12 (randomized between trials). **(B)** The participants were asked to convey the sequence's average, by vertically sliding a mouse-controlled bar set on a number ruler (white bar) between 1 and 100. The number corresponding to the bar's location is being dynamically displayed (30 in the display).

Four experiments were carried out to probe individual differences in the mechanism by which human observers evaluate rapid numerical sequences. In all the experiments the sequences presented two digit numbers at a rate of 4/s and sequence-length was randomly selected to be either 6 or 12 items (based on Brezis et al., 2015, we avoided shorter sequence lengths to discourage explicit computation strategies, which were found for sequences of 4 items). Previous research has indicated that the number magnitude is automatically encoded when subjects are presented with two digit numbers (Dehaene et al., 1990; Fitousi and Algom, 2019). In Experiments 1a and 1b, we require participants to evaluate only the sequence average (see Figure 3). These experiments differ in the distribution from which the numbers are sampled: uniform in 1a, and skewed in 1b. Under an adaptive observer assumption, skewed distributions are likely to make the deployment of a mid-range estimations less likely.^8^ In Experiments 2 and 3 we used (like in Experiment 1a) numbers that are sampled from a uniform distribution, but we required the participants to provide two estimates for each sequence. In addition to the average, Experiment 2 required an estimation of sequence-variance, while Experiment 3 required an estimation of the confidence in the estimation of the average. Since confidence is expected to reflect the uncertainty on the value of the estimate (Yeung and Summerfield, 2012; Lebreton et al., 2015) and this is likely to increase with the variance of the sequence, we consider the confidence estimation, an indirect/implicit estimation of the variance. Since mid-range (or min/max) strategies provide a simple and natural way to estimate the sequence-variance, we can expect that the deployment of this strategy will increase in Experiments 2 and 3 (see experimental methods for participants and procedure details for all experiments).

We thus manipulated two factors in our experiments: (I) the distribution from which the values are sampled (Experiment 1a vs. Experiment 1b), and the task complexity (estimating only the average; Exp 1 (a/b) or estimating both the average and the relative-variance (Experiment 2 and 3). Finally, the number of participants (*N* = 25) in all our experiments was selected based on previous studies that relied on model selection to classify participants to decision strategies (Newell and Shanks, 2003; Glöckner and Betsch, 2008; Pleskac et al., 2019).^9^

## Experiment 1a

### Methods

#### Participants

Twenty-five undergraduate from Tel-Aviv University (Mean age = 22.2; SD = 1.7) participated in the experiment. All participants were naive to the purpose of the experiment and had normal, or corrected-to-normal, vision. Informed consent was obtained from all subjects. Participants were awarded with course credit for their participation. All procedures and experimental protocols were approved by the ethics committee of the Psychology department of Tel Aviv University (Application 743/12). All experiments were carried out in accordance with the approved guidelines.

#### Apparatus and Stimuli

Displays were generated by an Intel I7 personal computer attached to a 24″ Asus 248qe monitor with a 144 Hz refresh rate, using 1920 × 1080 resolution graphics mode. Responses were collected via the computer mouse. Viewing distance was approximately 60 cm from the monitor. The stimulus consisted of sequences of 6 and 12 numerical values which were presented with a presentation rate of 4 HZ and were uniformly distributed. To generate the sequences, we used a 3 × 3 orthogonal design of mean: 40, 50, or 60 and ranges (to control variance) of mean ± 10, mean ± 25 or mean ± 39. The numbers in each sequence were independently sampled from one of these nine distributions (random between trials).^10^

#### Procedure and Design

Each trial began with a fixation display that consisted of a black 0.2° × 0.2° fixation cross (+) that remained on the screen for 250 ms. Then, a sequence of 6 or 12 numbers (randomized between trials) was presented. Once the sequence terminated, the participants were required to estimate the sequence's mean value using an analog ruler that was displayed and ranged from 1 to 100 (see Figure 3). Participants underwent 300 experimental trials divided into 10 blocks. Each block terminated with performance-feedback (real-average/estimated-average correlation) and a short, self-paced break.

### Results

We used two measures to quantify each subject's precision in averaging. The first is the Pearson correlation of the real and estimated averages of the sequences of each participant (see **Figure 6A**, for an example of an individual observer). The average Pearson correlation was high [*r*_(298)_ = 0.75, *SD* = 0.12] and was significantly higher than zero (all participants' *p's* < 0.001). Second, we computed the root mean square deviation (RMSD) between the real averages and the participants' responses (note that higher value of RMSD imply lower accuracy). The RMSD was significantly lower than the simulated RMSD generated by randomly shuffling participant's responses across trials [Actual RMSD = 7.7; Shuffled RMSD = 14.8; *t*_(24)_ = 18.7, *p* < 0.001, Cohen's *d* = 3.6].

**Figure 4:**
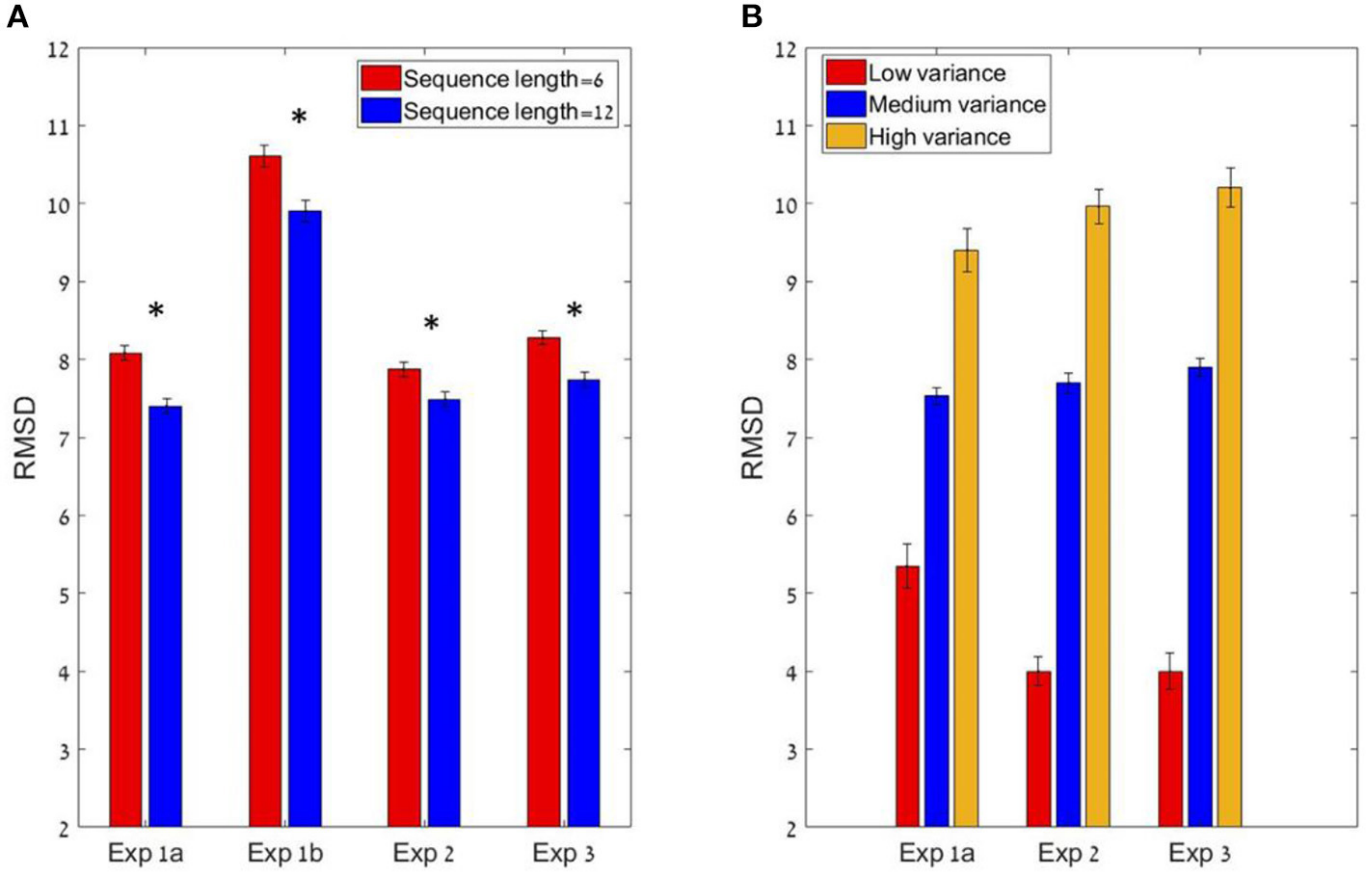
**(A)** The sequence length effect for all four experiments (* corresponds to a statistically significant effect). In all experiments, the participants performed significantly better (lower RMSD) in the 12 items condition compared to the six items (at group level). **(B)** The Variance effect for all experiments (excluding 1b) which did not manipulated variance. All 3 experiments showed a linear trend in which precision decreases as variance increases.

To test the sequence-length effect, we carried out a paired sample *t*-test between the RMSD of the six items condition compared to the 12 items condition, which showed a significant difference. The precision improved (RMSD decreased) with sequence-length: six items sequences (*Mean* = 8.08, *SD* = 3.3) and 12 items sequences (*M* = 7.4, *SD* = 2.9); *t*_(24)_ = 3.84; *p* < 0.01, Cohen's *d* = 0.77 (see Figure 4A). These results are consistent with the predictions of the normative-holistic model which predicts better performance for the 12 items sequences relative to the six items sequences due to the fact that uncorrelated single item noise averages out, but is also consistent with predictions of the mid-range model. In addition we found that precision decreases with sequence variance (see Figure 4B): repeated measure ANOVA with the within subject factor of the 3 groups of variance; *F*_(1.2,29.1)_ = 48.8; *p* < 0.001, $\eta_{p}^{2}$ = 0.67, indicating a linear trend.

**Figure 5:**
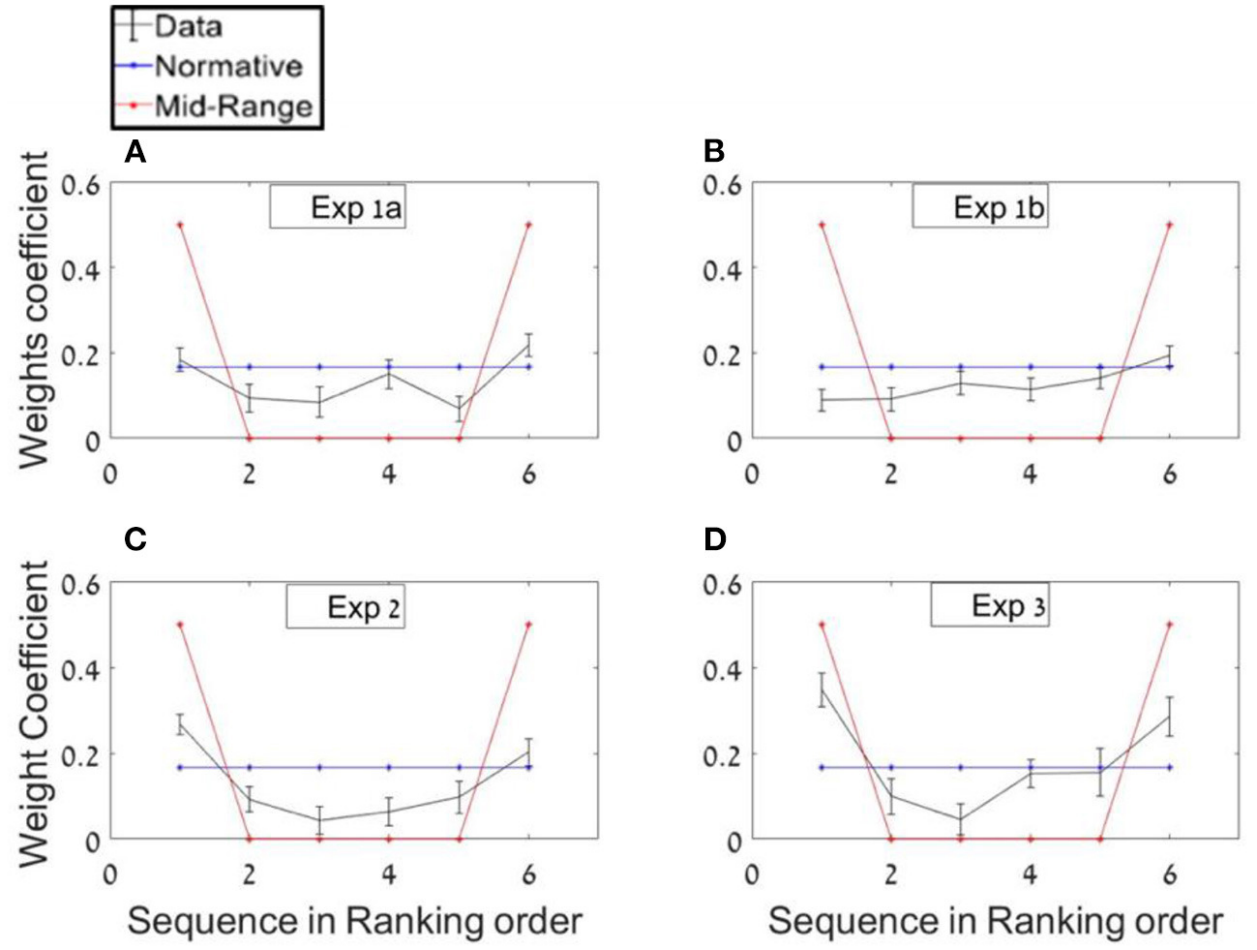
Ranking-weighting profile of the numbers **(A)** Experiment 1a. Real data regression (black) compared with normative-holistic and mid-range predictions (blue and red, respectively). **(B–D)** Same analysis for Experiments 1b, 2, and 3, respectively.

In order to probe whether participants based their average estimations mostly on extreme values, as predicted by a midrange strategy (see Figure 5, red plot), or on all items with equal weights as predicted by a normative-holistic strategy (see Figure 5, blue plot), we ran separate regressions for each of the sequences' length conditions, using the sequence ranked numbers as predictors (see Methods section; in the twelve-items sequences we paired the items in order to have six predictors in both conditions) and we averaged both conditions weights. As shown in Figure 5A, the regression weights show a mild U-shaped pattern (black line), indicating a tendency to overweight extreme values as predicted by a mid-range strategy, but note that the decision weights are closer to normative-holistic model (blue line), according to which participants based their estimations on all items. A paired sample *t*-test between the weight given to inlying ranks [2–5] and outlying ranks [1 and 6] (Vandormael et al., 2017) was statistically significant; *t*_(24)_ = 3.75, *p* < 0.001. The mean difference between outlying and inlying ranks across participants was 0.10.^11^

**Figure 6:**
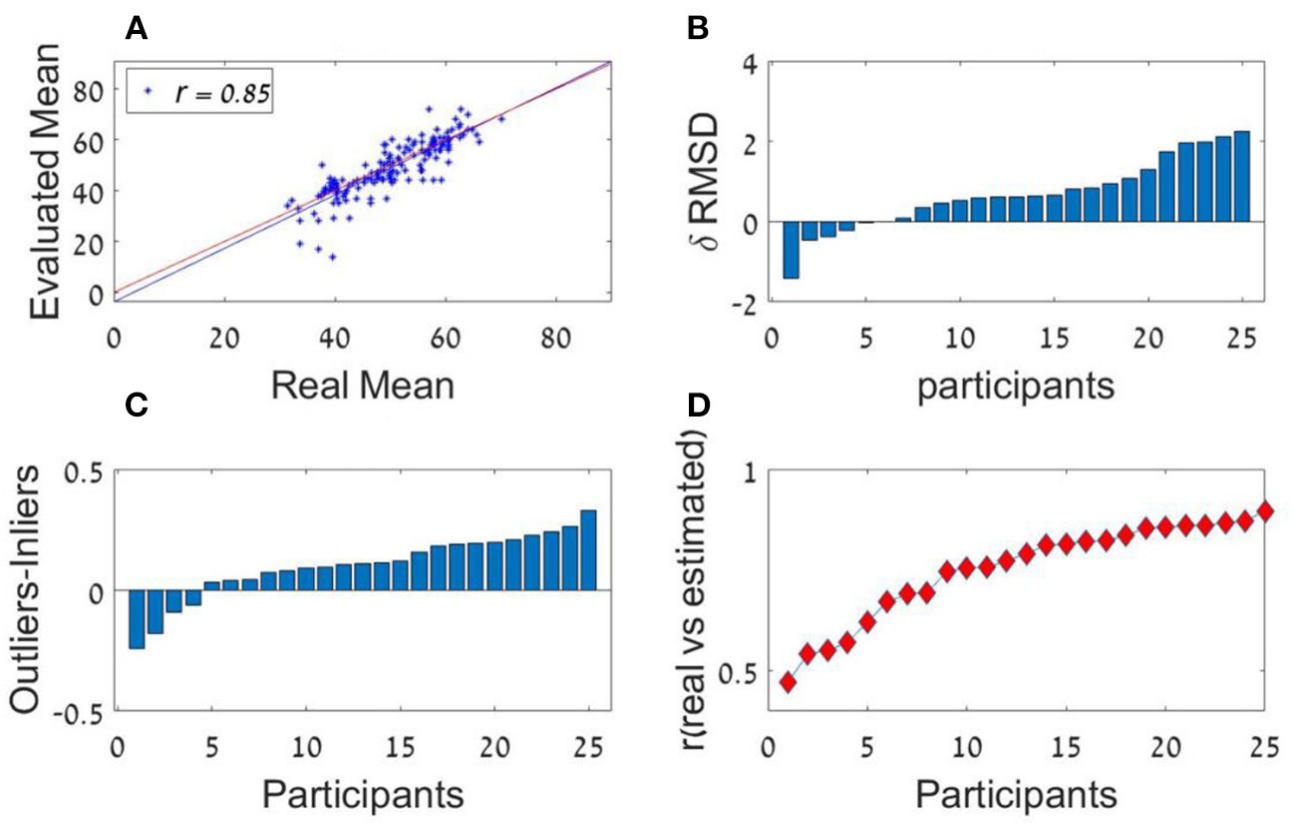
Experiment 1a **(A)** Trial by trial performance of a representative individual observer. The scatter plot depicts the participant's estimation (y-axis) for each of the presented number sequence averages (x-axis). Blue and red lines represent the regression and identity lines, respectively. **(B)** Individual differences in RMSD-difference (δ RMSD) between the two sequence length conditions (positive values indicate improvement in performance as sequence length increases; Participants are sorted by RMSD-difference (from the smallest from the smallest difference to the largest). **(C)** Individual differences in the difference between outliers' weight (the averaged coefficient of the first and last items in the sequence) and inliers' weight (the averaged coefficient of all items between the second and the one before last; sorted by outlier-inlier difference, from the smallest difference to the largest). **(D)** Individual differences in the correlations between the estimated average and the actual average (chance corresponds to *r* = 0). Participants in **(B–D)** are sorted independently.

We next examined individual differences. We found remarkable individual differences in the sequence-length effect (see Figure 6B) and in the curvature of the ranked decision weights (U-shape pattern), indicating the presence of different estimation strategies (Figure 6C). Remarkable individual differences in the precision of the estimations were also found (Figure 6D).

## Experiment 1b

Experiment 1b was identical to Experiment 1a, with the only exception that in the majority of trials the distributions used to generate the sequences were skewed rather than uniform (same means). In addition, the variance was not manipulated factorially, as in Experiment 1a, however, the trials had a high variability in their sequence mean and variance (see Methods section). Based on the adaptivity assumption, this was expected to reduce the reliance on the mid-range strategy (see footnote 8), as indicated in flatter ranked regression weights (closer to the blue than the red-line in Figure 5B).

### Methods

#### Participants

Twenty-five undergraduate from Tel-Aviv University (Mean age = 22.5; *SD* = 2.3) participated in the experiment. All participants were naive to the purpose of the experiment and had normal, or corrected-to-normal, vision. Informed consent was obtained from all subjects. Participants were awarded with course credit for their participation. All procedures and experimental protocols were approved by the ethics committee of the Psychology department of Tel Aviv University (Application 743/12). All experiments were carried out in accordance with the approved guidelines.

#### Stimulus Materials and Procedure

**Figure 7:**
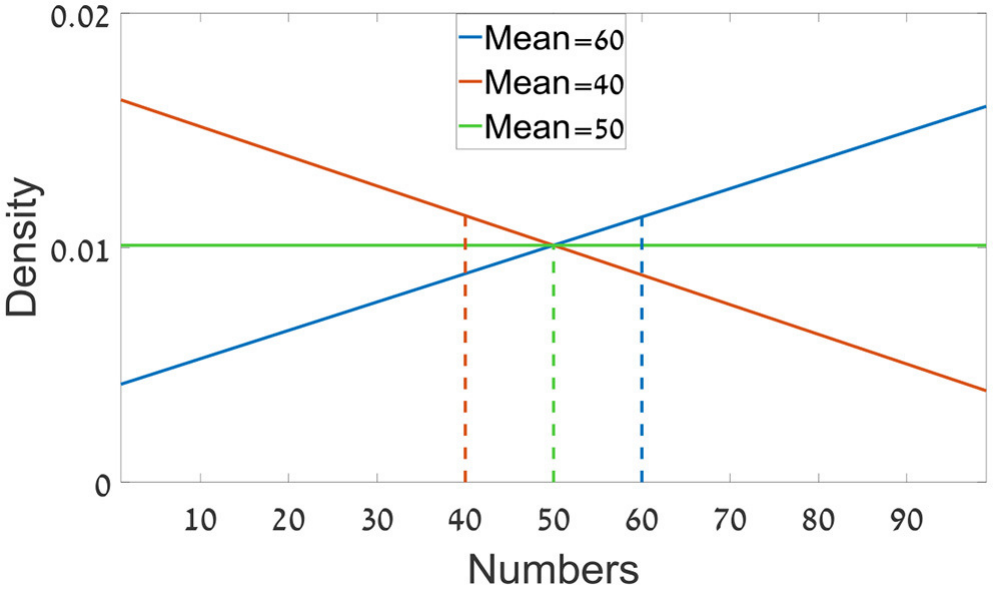
The distributions from which the numbers were sampled in Experiment 1b.

The only difference from Experiment 1a was that the sequences in each trial were sampled from predefined three distributions ranged between 1 and 99; with means of: 40, 50, or 60. Two of the distributions were triangular skewed density distributions (one from each side), and the third one was a uniform distribution (Figure 7). Each sequence was sampled independently from one of the three distributions.

### Results

Participants showed a high accuracy rate in evaluating sequences' average in both lengths. Pearson mean correlation between the sequences' actual average and participants' estimation of the average was high [*r*_(298)_ = 0.71, *SD* = 0.12] and significantly higher than zero (all *p's* < 0.001). In addition, RMSD between the real averages and participants' responses was also measured and was significantly lower than simulated RMSD generated by randomly shuffling participants' responses across trials; RMSD = 10.3, RMSD random Shuffle = 18.7; *t*_(24)_ = −20.5 *p* < 0.001, Cohen's *d* = 4.23. Just like Experiment 1a, there was a significant decrement in RMSD with sequence-length (see Figure 4A Experiment 1b; 6 items sequences (M = 10.6, SD = 2.5) and 12 items sequences (M = 9.9, SD = 2.5) trials; *t*_(24)_ = 2.88, *p* < 0.01, Cohen's *d* = 0.57. A repeated measures ANOVA revealed that there is no distribution effect on RMSD; *F*_(2,48)_ = 0.95; *p* = 0.39.

Due to the fact that in this experiment the numbers were selected from skewed distributions (in most of the trials), we expected participants to rely less on extreme values which could mislead them in average estimation. In order to test this adaptivity hypothesis, the same ranking analysis from Experiment 1a was used in the current experiment. Consistent with the adaptivity hypothesis, and unlike in Experiment 1a, in Experiment 2 no significant differences between in/out-lying ranking weights was found; *t*_(24)_ = 1.14, *p* = 0.3 (see Figure 5B).

## Experiment 2

Experiment 2 was identical to Experiment 1a with the exception that in addition to evaluating the sequences' average, participants were also instructed to estimate the sequences' relative “spread”^12^ (high, medium, low). The experiment had two aims: First, we wanted to see if people have the ability to extract this higher order statistic, together with the average, when viewing rapid sequences of numbers. Second, we wanted to see if introducing this demand, would change the strategy the participants deploy to estimate the average.

### Methods

#### Participants

Twenty-five undergraduate from Tel-Aviv University (Mean age = 23.3; SD = 2.7) participated in the experiment. All participants were naive to the purpose of the experiment and had normal, or corrected-to-normal, vision. Informed consent was obtained from all subjects. Participants were awarded with course credit for their participation. All procedures and experimental protocols were approved by the ethics committee of the Psychology department of Tel Aviv University (Application 743/12). All experiments were carried out in accordance with the approved guidelines.

#### Stimulus Materials and Procedure

Same as in Experiment 1a, but after evaluating the average using the numbers ruler, participants were asked to evaluate the “spread of the sequence” on a 3-category scale: (i) small, (ii) medium, (iii) large (the participants were shown 2 example sequences of each kind at the beginning of the experiment).

### Results

**Figure 8:**
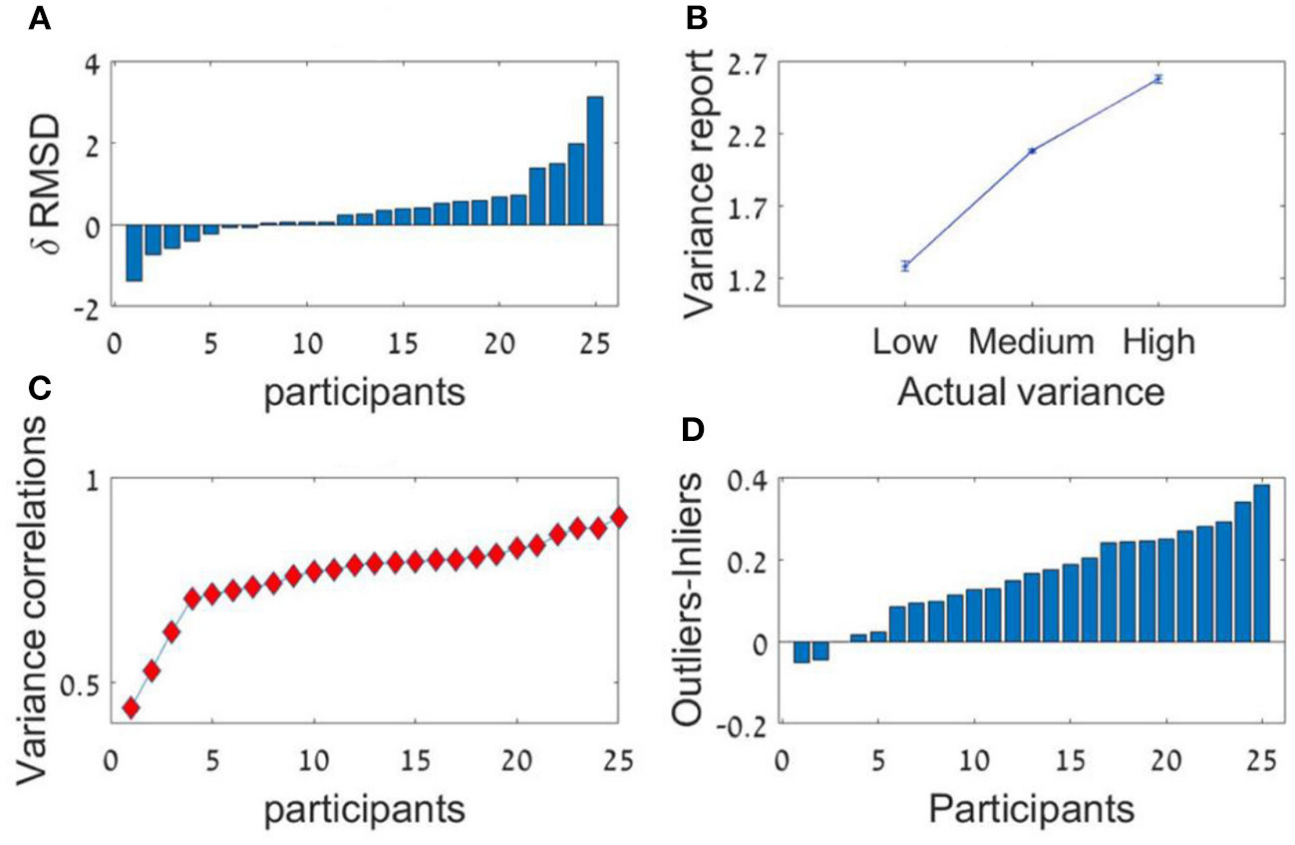
Experiment 2 **(A)** Individual differences in δRMSD between the sequence length conditions (positive values indicate improvement in performance as sequence length increases. **(B)** The average variance reported (y-axis) as a function of the real variance of the sequence (x-axis). **(C)** Individual difference in the Spearman correlation between variance reported and actual variance for each participant. **(D)** Individual differences in δ between outliers' weight (the averaged coefficient of the first and last items in the sequence) and inliers weight (the averaged coefficient of all items between the second and the one before last).

The participants had a high accuracy rate in evaluating sequences' average in both lengths. Pearson mean correlation across participants between the sequences' actual average and participants' estimation of the average was high [*r*_(298)_ = 0.73, *SD* = 0.09] and significantly higher than zero (all *p's* < 0.001). In addition, the RMSD between the real averages and participants' responses was significantly lower than simulated RMSD generated by randomly shuffling participants' responses across trials [RMSD = 7.69, RMSD random Shuffle = 14.23; *t*_(24)_ = −23.82, *p* < 0.001, Cohen's *d* = 4.8]. Similarly to previous experiments, there was a significant decrement in RMSD with sequence-length [see Figure 4A, Experiment 2; *t*_(24)_ = 2.1, *p* < 0.05, Cohen's *d* = 0.42], however it was subject to marked individual differences (Figure 8A).

Critically, the participants showed a high accuracy rate in the estimation of the sequences' relative-variance in both lengths (Figure 8B). Spearman correlation between the sequences' actual variance and participants' (3 point-scale) estimation of the relative-variance was high and significant [*r*_(298)_ = 0.74, *SD* = 0.1, *p* < 0.001; for all participants; Figure 8C].

As in Experiment 1 we averaged both regressions weights of each of the sequences' length conditions, using the sequence ranking's numbers as predictors. The results show a U-shaped pattern with a significant difference between the decision weights of the inliers [ranks 2–5] and outliers ranked numbers [ranks 1 and 6]; *t*_(24)_ = 6.78, *p* < 0.001. The mean difference between outlying and inlying ranks across participants was 0.16. The higher difference in this experiment compared with Experiment 1a (0.1) indicates that instructing the participants to estimate the variance in addition to the average, made them assign even higher weights to the extreme values (Figure 5C). This was also subject to individual differences (Figure 8D).

## Experiment 3

Experiment 3 was similar to Experiment 2, but here we replaced the explicit estimation of the sequence relative-variance with a more implicit one—a report of confidence in the average estimation (we expected that confidence will decrease with sequence variance; (Yeung and Summerfield, 2012; Lebreton et al., 2015). Furthermore, we aimed here to measure the decision time for the average-estimation and to determine if it depends on sequence variance (Ratcliff and McKoon, 2020).

### Methods

#### Participants

Twenty-five undergraduate from Tel-Aviv University (Mean age = 22.4; SD = 2.7) participated in the experiment. All participants were naive to the purpose of the experiment and had normal, or corrected-to-normal, vision. Informed consent was obtained from all subjects. Participants were awarded with course credit for their participation. All procedures and experimental protocols were approved by the ethics committee of the Psychology department of Tel Aviv University (Application 743/12). All experiments were carried out in accordance with the approved guidelines.

#### Stimulus Materials and Procedure

**Figure 9:**
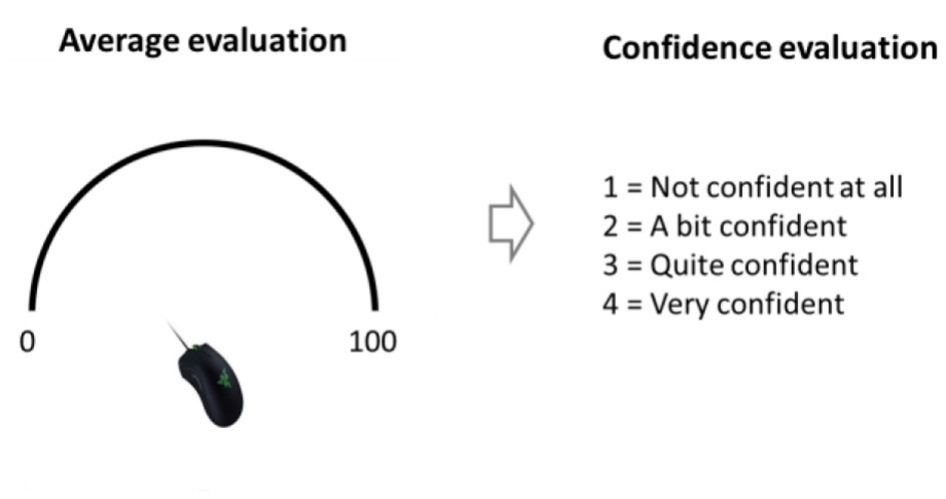
Response scale and confidence response in Experiment 3.

Experiment 3 was similar to Experiment 2 with three exceptions: (i) In addition to the 9 distributions that the sequences were sampled from in the previous experiments, there was one more condition with bimodal sequences (mean of 50 in which half of the numbers were sampled from the uniform distribution: *U* (5,35) and the other half from *U* (65, 95). (ii) The average estimation was made using a semicircle shaped scale. The mouse cursor was always at the middle of the circle at the beginning of the scale display, thus it had an equal distance from each point of the scale (see Figure 9). The reason we changed the scale from a ruler to a semicircle was so we could measure more precisely the reaction time of the average estimation without introducing anchoring bias. (iii) Instead of evaluating the spread of the sequence, after evaluating the average, the participants were instructed to rate their confidence rate on a 1–4 scale.

### Results

The participants had a high accuracy in evaluating the sequences' average as shown by a high Pearson correlation between real and estimated averages [*r*_(298)_ = 0.72, *SD* = 0.09, all *p's* < 0.001]. In addition, the precision of the estimation was significantly higher (RMSD was lower) between the real averages and participants' responses compared with that obtained by randomly shuffling participants' responses across trials [RMSD = 8.0, RMSD random Shuffle=14.3; *t*_(24)_ = −24.08*, p* < 0.001, Cohen's *d* = 5.18].

**Figure 10:**
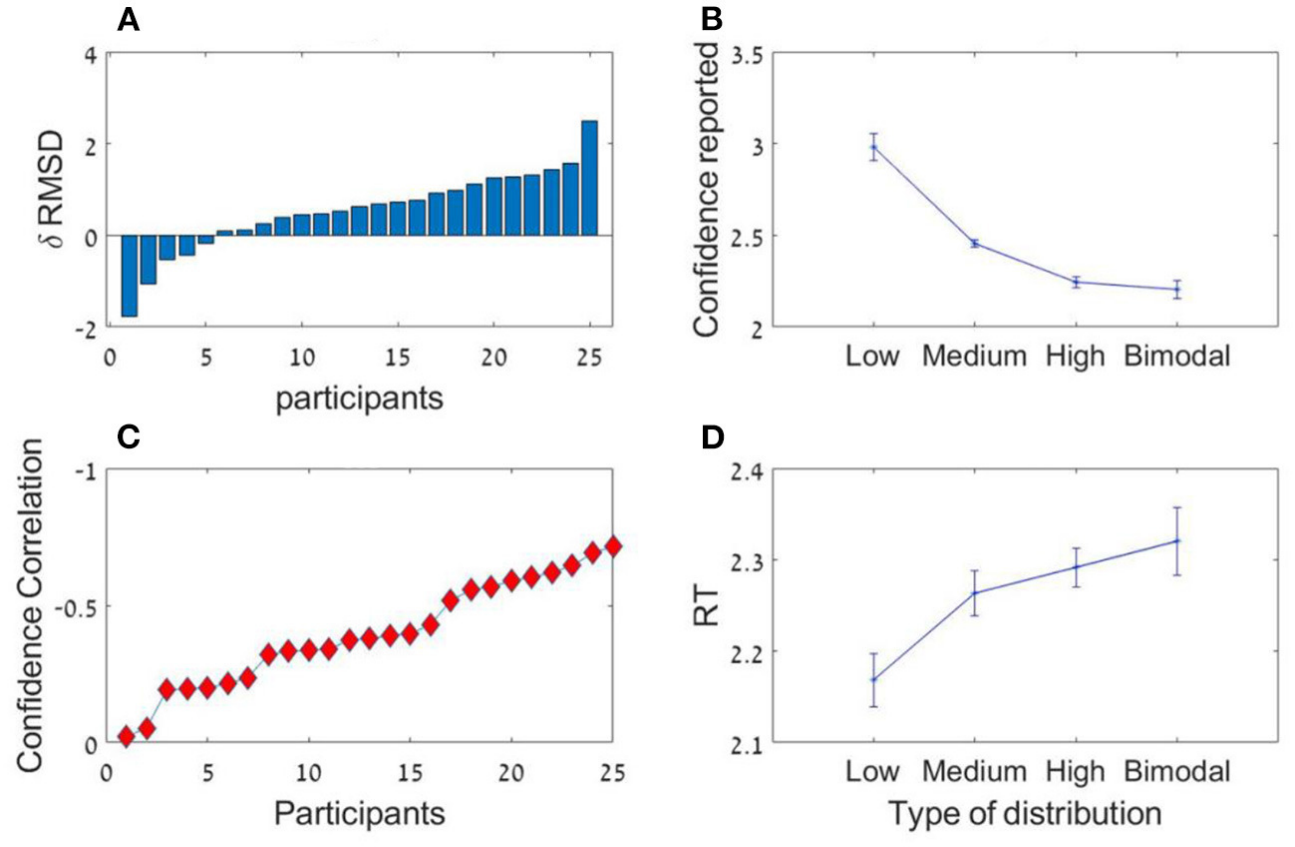
Experiment 3 **(A)** Individual differences in δ RMSD between the sequence length conditions (positive values indicate improvement in performance as sequence length increases. **(B)** Confidence reported as a function of variance. **(C)** Spearman correlations between variance and confidence rate for each participant. **(D)** RT as a function of variance.

As in the previous experiments, there was a significant effect for sequence length in the RMSD indicating better performance in 12 items sequences *t*_(24)_ = 3.0; *p* < 0.01, Cohen's *d* = 0.6, which was subject to marked individual differences; see Figure 10A). Critically, the confidence reported by the participants decreased with the sequence variance; *F*_(1.2,30.1)_ = 43.92; *p* < 0.001, $\eta_{p}^{2}$ = 0.64 (Figure 10B). The Spearman correlation (for each participant across all responses) between the sequences' variance and the participants' (4-scale) confidence report was significant [*r*_(298)_ = −0.38, *SD* = 0.18, *p* < 0.001], but subject to marked individual difference (Figure 10C). Finally, we found that the RT of the average estimation increased with the sequence variance; *F*_(3,72)_ = 3.9; *p* < 0.05, $\eta_{p}^{2}$ = 0.14. For the novel (bimodal) condition we found that the confidence and the RT were similar to those in the (unimodal) large variance condition (Figure 10D).

Finally, we ran a ranking regression to test whether participants assign higher weights to extreme values. As in the previous experiments a paired *t*-test indicated an over-weighting of the extreme values, *t*_(24)_ = 4.27, *p* < 0.001. Mean difference between the outlying and inlying ranks was 0.20 (see Figure 5D).

## Summary of Experimental Results

The results of these experiments demonstrate that human observers have a high capacity to extract two major summary statistics—the average and the relative variance, and to evaluate them with a relatively high accuracy when viewing rapid numerical sequences presented at a rate of 4/s. One novel aspect of the results is the accurate estimation of sequence relative variance (Figures 8B,C), and the fact that confidence and RT in the average-estimation can be used as indirect measures of sequence variance (Figures 10B,C).

The results also indicate significant variability in the strategy that the observers deploy in these estimations. This variability is indicated in the variation of the average-estimation precision, in the variation of the sequence length effect and the curvature of the U-shape in the decision weights for ranked sequence elements (Figure 5). As all of these factors distinguish between the strategies we labeled as holistic-normative vs. mid-range, it is likely that they reflect a variability in the strategy the participants deploy. In particular, it may be suggested, that type of distribution from which the numbers are sampled (see differences between Experiments 1a and 1b in the ranked regression) and the need to monitor variance (see differences between Experiments 1 and 2), affects the likelihood of participants to deploy a mid-range vs. a holistic normative estimation.

In order to quantify and validate these qualitative summaries of the data, we have carried out a computational analysis, aimed to contrast between the averaging estimation strategies we considered earlier (holistic-normative vs. mid-range). In the following section, we use computational modeling methods to classify participants based on which of these strategies they deployed in each task. We wish to clarify that we do not see these classes of strategies as exhaustive for the task. However, they are the simplest we could think of, that illustrate each of the types of estimation we aimed to focus on, and which have the needed features to account for the variability obtained in our data.

## Individual Differences in the Average-Estimation Strategy: Strategy Classification

To classify the averaging-estimation strategies, we used two complementary approaches. First, we classified a subject as using a holistic-normative or a midrange strategy on the basis of the highest correlation (across all the trials the participant did) between the actual response and the true average (or midrange) of the sequences in that trial. While this method is simple, it only relies on the average of the estimation and does not take the distribution of estimations into account. In the second approach, we developed formal models that quantify the predictions of the holistic and of the mid-range strategies, under a number of assumptions. As these models include stochastic processing, they predict not only mean-estimation but full distribution of estimations, and thus we can compute for each trial the likelihood of the response given the model and its parameters (Note that once we determine the noise sources, the models also make predictions for the RMSD as a function of sequence-length, and thus this analysis goes beyond what could be achieved via a regression approach).

For each type of model (holistic or mid-range) we examined two versions, a simple one in which the noise (variability) parameters are fixed, and a more complex one in which one variability parameter depends on the sequence-variance. The simpler models assume the presence of a common motor/output noise (that is invariant to sequence-length or sequence-variance) and an encoding noise in the holistic model, which operates in the conversion of each item from a numerical symbol to a population response over magnitude representations (Dehaene, 2007; see also Figure 1^13^; both of these noise distributions are assumed to be Gaussian. In the simple midrange model we assumed that the symbolic calculation of the average of two numbers is accurate and does not depend on their range; but see Complex-models below for a model classification that does not make this assumption).

In the complex models, variance dependent noise was introduced for both model types. This is motivated by this feature of the data (Figure 4B) and can be motivated in different ways for the two types of models. For the holistic model, the variance dependent variability is motivated by the holistic population-averaging model (Brezis et al., 2018), in which the estimation of the center of mass of the population response depends on the variance of the sequence (peaked distributions are more precise than flat ones; Figure 1C). For the mid-range model, the variance dependent noise corresponds to a type of “calculation-error,” which increases with the range. Finally, in all models we allowed an intercept (*a*) and a slope (*b*) to mediate the transformation from an internal to an external space^14^ (see section Computational Methods for details of the models). For each participant, we have carried out a model selection analysis of the data in the experiments presented. The models which we contrasted were the normative-holistic model and the mid-range model. The contrast was based on their relative likelihood to account to all the estimations a participant gave (maximum likelihood, subject to a standard penalty for degrees of freedom; *AIC* and *BIC* measures, Akaike, 1973; Schwarz, 1978; Kass and Raftery, 1995).

## Computational Methods

To apply the models to data, we had to make some assumptions on the response variability in these 4 models. For the simple normative-holistic model we have 2 noise parameters, an encoding noise^15^ (which is applied for each item, and thus averages out as $1/\sqrt{2}$ with sequence length, from 6 to 12) and a global (or motor noise^16^), which does not change with sequence length:


(3) $$\begin{array}{l} MeanEstimated=a+b\left(\frac{\sum_{i=1}^{n}X_{i}+\varepsilon_{e}}{n}\right)+\varepsilon_{m},\varepsilon_{e}\\ \;\sim N\left(0,\sigma_{e}^{2}\right)\;and\varepsilon_{m}\sim N\left(0,\sigma_{m}^{2}\right) \end{array}$$

where, *x*_*i*_ is the *i*th item of the sequence, ε_*e*_ is the encoding noise and ε_*m*_ is the motor noise and *a* and *b* are intercept and slope parameters, respectively, that are used to map between the participant internal estimation to the external response scale.

For the more complex normative-holistic model we have three noise parameters. Additional to the motor noise (as before), the encoding now varies with the sequence-variance (two parameters instead of one):


(4) $$\begin{array}{l} MeanEstiamated=a+b\left(\frac{\sum_{i=1}^{n}X_{i}+\varepsilon_{e+}{\varepsilon_{v}\cdot \sigma }_{s}^{2}}{n}\right)+\varepsilon_{m},\varepsilon_{e}\;\\ \;\sim N\left(0,\sigma_{e}^{2}\right),\;\varepsilon_{v}\;\sim N\left(0,\sigma_{v}^{2}\right) \end{array}$$

and $\varepsilon_{m}\;\sim N\left(0,\sigma_{m}^{2}\;\right)$

where, x_i_ is the ith item of the sequence, ε_*e*_ and ε_*v*_ are encoding noise parameters, ε_*m*_ is the motor noise and $\sigma_{s}^{2}$ is the sequence's variance. In principle, this model could be extended to consider potential *leak* of information, during the sequence presentation (Usher and McClelland, 2001; see also Katzin et al., 2021 for a model using leak in the averaging of numerosity sequences). Since recency effects in this experimental paradigm are relatively small (especially for high list length sequences; see Brezis et al., 2015), we decided (for the sake of simplicity) to not introduce this component in our set of models.

In the simple mid-range model we only assumed the presence of global/motor noise, which does not depend on sequence length:


(5) $$\begin{array}{l} MeanEstimated=a+b\left(\frac{x_{\max}+x_{\min}}{2}\right)+\varepsilon_{m},\varepsilon_{m}\sim N\left(0,\sigma_{m}^{2}\right) \end{array}$$

where a is the intercept, b is the slope, *x*_max_ and *x*_min_ are the smallest and highest items of the sequence and ε_*m*_ is the motor noise.

In the complex mid-range model we assumed a ‘calculation’ noise which depends on the range of the sequence in addition to the global/motor noise, which does not depend on sequence length:


(6) $$\begin{array}{l} MeanEstimated=a+b\left(\frac{x_{\max}+x_{\min}}{2}+\varepsilon_{c}\frac{x_{\max}-x_{\min}}{2}\right)\\ \;+\varepsilon_{m},\varepsilon_{m}\sim N\left(0,\sigma_{m}^{2}\right)\wedge \varepsilon_{c}\sim N\left(0,\sigma_{c}^{2}\right) \end{array}$$

where a is the intercept, b is the slope, *x*_max_ and *x*_min_ are the smallest and highest items of the sequence, ε_*m*_ is the motor noise and ε_*c*_ is the calculation noise.

Thus, the simple holistic-normative model has four free parameters (slope, intercept, encoding/motor noises), while the simple mid-range model has three free parameters (slope, intercept, motor noise). For the complex models we added one more parameter for each model to allow variance dependent noise. These noise components are motivated by the holistic population-averaging model (Brezis et al., 2018) in which the estimation of the center of mass of the population response depends on the variance of the sequence (peaked distributions are more precise than flat ones; Figure 1C). For the mid-range model, the variance dependent noise corresponds to a type of “calculation-error,” which increases with the range. For each trial in the experiment, we analytically computed the probability distribution for an estimation (x) as a function of the sequence of numbers the participants received on that trial and the model parameters, and we collected the Log of the Probability for the response on that trial given model parameters. This Log-Probability was aggregated across all the trials, and we searched the model parameter space (using a combination of grid and Simplex; see Optimization procedure in Supplementary Material) for the set of parameters with highest log-likelihood. The model selection was then based on maximum likelihood model fits, and for each participant the model with the lowest AIC/BIC was selected. Before carrying out the model classification on the data we have carried out a model classification recovery, in which we generated synthetic data (based on each of the model types with parameters in the range found from in the data fits) and we observed a high classification recovery (98% correct classifications; see Supplementary Material).

## Models Fits and Model Comparison

**Figure 11:**
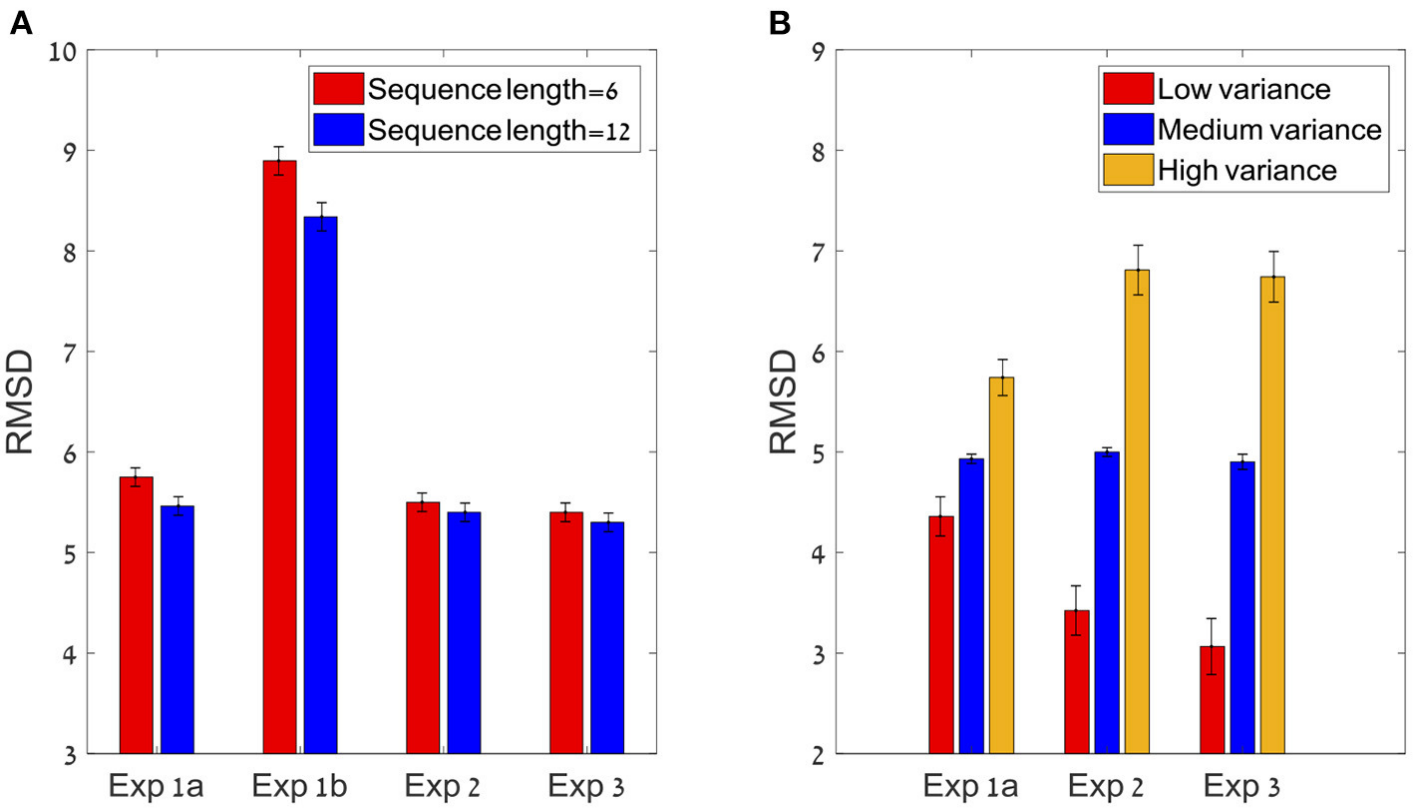
Models' best fit account of behavioral patterns. In order to test whether the models account for the sequence length and variance effect (see Figures 4A,B), we simulated the best fit predictions in each experiment (for the variance effect we simulated only experiments 1a, 2, and 3 in which variance was manipulated). **(A)** Models prediction to the sequence length effect. **(B)** Models prediction to the variance effect in experiment 1a, 2, and 3. All predictions are based on best fit model (and parameters) for each participant.

In order to test whether the models accounted well for the qualitative behavioral patterns in our experiments: the dependency of the precision-estimate (RMSD) on the sequence-length and on sequence-variance, we simulated responses using the winning model optimized parameters for each participant (see Figure 11). The model parameters of these “best fit models” are presented in Supplementary Figures 2–9. Note in particular that the mean b-parameter are in the range (0.5–1); for example, the average-b for the simple holistic model is 0.77 (SE of 0.012), indicating a moderate degree of regression to the mean, as observed in the data (Figure 6A).

As in the data (see Figure 4A), the sequence length effect was bigger in Experiments 1a and 1b (in which most participants were classified as holistic), compared to Experiments 2–3 (only about half of the participants holistic). This can be explained by the stronger sequence-length dependency in this model (see Figure 2).

In addition, we simulated the best fit of each model in order to contrast model-predictions. The simulation showed that the mid-range model failed to predict a sequence-length effect (see Supplementary Figure 1).

In Table 1, we present the group BIC-values (mean BIC across participants) for each of the four models for the data of our four experiments. At the group level, the holistic model wins by far in Experiments 1a and 1b, in which subjects where only asked to estimate the sequence-average. In Experiments 2 and 3 (in which variance or confidence where queried in addition) we find more balanced results; there is a small advantage favoring the mid-range model in both experiments. We also see, that except for Experiment 1b (which used skewed number distributions), the presence of variance-noise improved the model fits.

**Table 1:** Average BIC value across participants for each model in each experiment (bold values mark the winning model at each level: simple/complex).

**Experiment**	**Simple**	**Simple**	**Holistic**	**Mid-range**
	**holistic**	**midrange**	**with variance**	**with variance**
Experiment 1a	**1,995**	2,031	**1,961**	1,982
Experiment 1b	**2,157**	2,246	**2,160**	2,247
Experiment 2	**1,983**	1,996	1,948	**1,947**
Experiment 3	**2,021**	2,036	1,981	**1,976**

The result of the strategy classification in the four experiments is summarized in Table 2, based on BIC-measures (see Supplementary Material for the tables with AIC/BIC fit measures of individual participants) for the simple model variants and in Table 3 for the complex model variants.

**Table 2:** Number of participants (out of 25) classified as using the simple holistic vs. mid-range in their average estimations, in the four experiments based on BIC values.

**Experiment**	**Simple holistic**	**Simple mid-range**	**Percentage holistic (%)**
Experiment 1a	23 (24)	2 (1)	92
Experiment 1b	25 (25)	0 (0)	100
Experiment 2	16 (17)	9 (8)	64
Experiment 3	20 (20)	5 (5)	80

*Values in parentheses correspond to the classification based on the empirical correlations (see text)*.

**Table 3:** Number of participants (out of 25) classified as using the complex holistic vs. mid-range in their average estimations, in the four experiments based on BIC values.

**Experiment**	**Complex-holistic**	**Complex**	**Percentage**
		**midrange**	**holistic (%)**
Experiment 1a	22	3	88
Experiment 1b	25	0	100
Experiment 2	12	13	48
Experiment 3	12	13	48

First, we see a very high consistency between the simple-model classification and that based on empirical (trial by trial) correlations between the models and the subject's estimates. Second, based on both (simple/complex) model types, we see that almost all subjects are classified as deploying a holistic estimation mechanism in Experiments 1a and 1b (only 2–3 out of 50 s were classified as mid-range in these experiments).^17^ Consistent with the group data, we also find that about half of the participants still use the holistic estimation in Experiments 2 and 3, when they are required to estimate not only the sequence-average but also its relative-variance (or to report their confidence), but we also see that the requirement to report variance or confidence leads to an increase in the fraction of participants who deploy midrange strategies.^18^

The classification results are also consistent with the ranked regression results (Figure 5), where we see a gradation in the fraction of participants that deploy, a holistic/normative vs. a mid-range strategy, respectively. While in Experiment 1b, which presented numbers selected from skewed distributions, all the participants were classified as holistic/normative and they showed flat ranked regression weights, in all other experiments, there were some participants classified as mid-range.^19^ Moreover, we see that the fraction of participants using a mid-range strategy increases (and the fraction of those using a holistic estimation decreases) when the participants are required to monitor the sequence variance or the confidence in the average estimation. This is consistent with the increased weights given to the extreme values at the group level, in these two conditions and with the marked variability in the sequence-length effect (and its reduced effect magnitude in Experiments 2 and 3).

**Figure 12:**
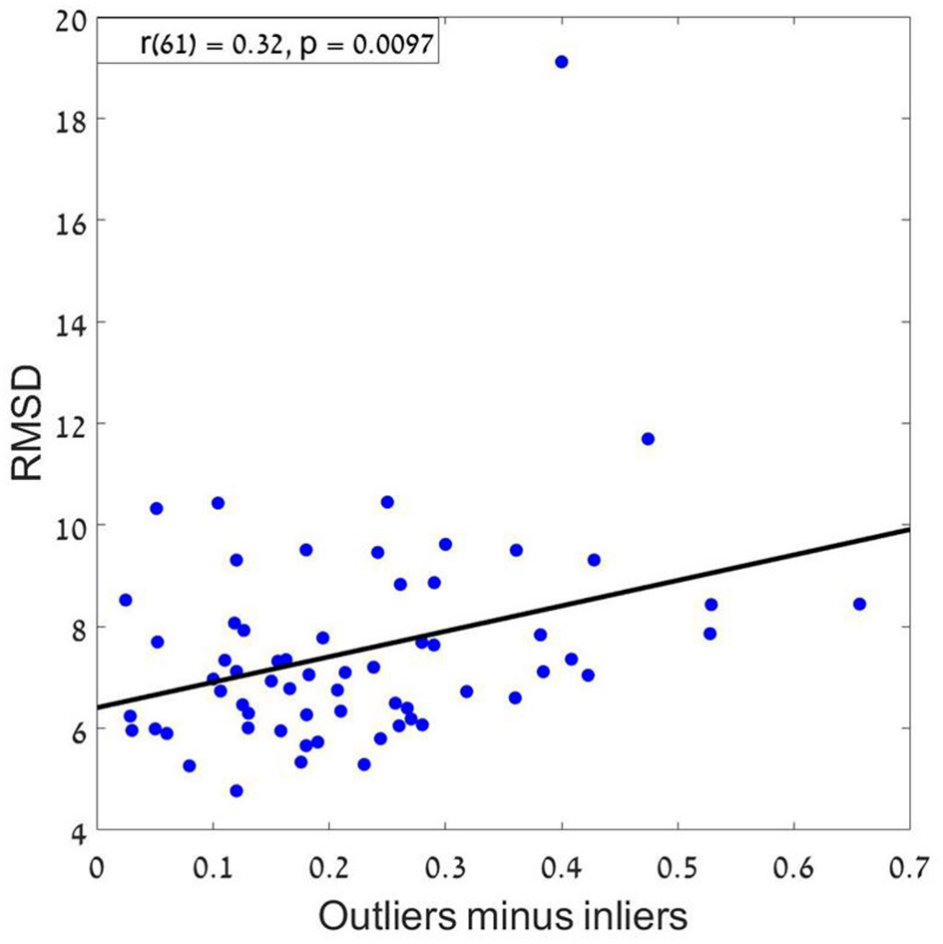
RMSD as a function of the difference between outliers and inliers. A positive correlation means that as the difference increase (more weights to the outliers) the RMSD increases, resulting with a bigger error.

Finally, we tested whether estimation precision (RMSD) differs between participants who were classified to the holistic vs. mid-range (this was done only for Experiments 2–3, where there roughly an equal number of participants were classified for both models).^20^ An un paired *t*-test indicates that holistic subjects were significantly more precise than mid-range subjects (Mean Values (SE) are, 7.2 (0.38) and 8.4 (0.3), for the subjects classified as holistic vs. mid-range, *t*_(47)_ = 2.26, *p* < 0.05, in their sequence-average estimations. To further support this conclusion, we also ran a correlation analysis by correlating the difference between outliers' weight (the averaged coefficient of the first and last items in the sequence) and inliers weight (the averaged coefficient of all items between the second and the one before last) with RMSD. The results showed a significant positive correlation (*r* = 0.32; *p* < 0.01) which supports our conclusion from the previous analysis, that a holistic strategy results in better performance compared to mid-range strategy^21^ (see Figure 12).

## Discussion

In four experiments we have shown that human observers can make quite accurate estimations of the average of rapid sequences of two digit numbers presented at a rate of 4/s (as quantified by Pearson correlations in the range of 0.7–0.8, between actual and estimated averages). Moreover, we show that the observers are able to estimate, in addition, the relative variance of the sequence (high, medium or low): the average Spearman correlation between estimated and actual (relative) variance was 0.74 (see Figure 8C). Furthermore, the observers speed and confidence in the estimation of the average decreased with the variance of the sequence (Figure 10). These results demonstrate that the remarkable ability to extract summary statistics of perceptual sets (Ariely, 2001; Dakin, 2001; Parkes et al., 2001; Chong and Treisman, 2005; Haberman and Whitney, 2011; Bronfman et al., 2015), extends to rapid numerical sequences.

A central question that these results pose is the nature of the mechanism or strategy that the observers deploy to generate these estimates. Due to the rapid sequence presentation and the strict response-deadline we impose on response generation (3 s, including the response on a continuous scale), we can exclude a fully analytical/symbolic strategy of adding the numbers and dividing by the sequence length, in favor of more intuitive estimation strategies. Two such “intuitive” strategies were considered in detail here. The first is the estimation of the center of mass from a noisy holistic (or frequency based) representation of each sequence, and the second is an estimation based on few (efficiently selected) samples. For the former we have shown it to predict an improvement in precision with sequence-length and for the latter we distinguished between random sampling, which predicts a decreasing precision with sequence length, and a mid-range strategy which predicts a milder improvement (Figure 2). Since most of the subjects in all experiments (and three out of our four experiments at the group level) show an improvement in precision with sequence-length (see Figure 4A), this rules out the random sampling as an account for task performance in our experiments, leaving the holistic (or frequency) model and the mid-range strategy, as the more plausible candidates.

The most distinctive signature of the mid-range strategy (compared with the holistic/normative model) is the U-shape pattern in the decision weights of the ranked samples (Spitzer et al., 2017; Vanunu et al., 2019, 2020). In all our experiments, except the one which utilized skewed distribution of numbers (experiment 1b), we found a small U-shaped modulation at the group level (Figure 5), which was subject to marked individual differences (Figures 6C, 8D). Model classification of individual subjects confirmed the presence of individual differences. While all the subjects were classified as utilizing the holistic mechanism in Experiment 1b (skewed distributions), we found that 12% were classified to utilize mid-range in Experiment 1a (uniform distribution), where this strategy is a viable one for the task. Whereas, this comparison needs to be qualified by the relatively low number of participants (*N* = 25 per group), it does support the idea that the averaging mechanism is sensitive to the distribution from which the values to be averaged are sampled, which is likely to modulate value expectations and gain modulations (Summerfield and De Lange, 2014). In particular, since averaging values sampled from skewed distributions results in higher deviations between the true-average and the mid-range, a shift from the mid-range strategy implies adaptivity of the participants to task contingencies. Follow up studies with larger number of participants^22^ will be needed, however, to further establish this conclusion.

Furthermore, the fraction of subjects that appear to utilize the mid-range strategy appears to increase in Experiments 2–3 (54%), in which the participants were asked to estimate the sequence relative spread or their confidence in the average estimation. Note that this comparison groups Exp. 1A and 1B together (no evaluation of spread required) and Exp. 2–3 together (evaluation of the spread required) to obtain a larger sample (of *N* = 50) per group. This shift implies that the introduction of a secondary estimate (relative spread or confidence) increases the chance that participants will rely on a strategy that monitors the min and the max of the sequence (Myczek and Simons, 2008), as this provides for free an estimate of the relative-spread (in terms of the sequence-range). Similarly, subjects could rely on range estimations as a proxy for confidence. Nevertheless, even in Exp. 2–3, about half of the participants appear to rely on a holistic estimation, indicating that the monitoring of the min and max of a sequence is not the only way to estimate the variance and the confidence in the average estimate, and that a holistic process (which does not monitor the min or the max) is also viable. These results parallel those obtained in tasks of probabilistic inference or in multiatribute decisions, in which the majority of participants rely on a holistic/automatic^23^ compensatory strategy of evaluation, while a minority rely on *Take the Best* (Newell and Shanks, 2003; Glöckner and Betsch, 2008; Betsch and Glöckner, 2010; Brusovansky et al., 2018).

Although we found that most observers deployed the holistic estimation mechanism, both estimation models can be subject to variations. For example, one can modify the holistic model so as to give differential attentional weights to numbers based on their values or their temporal order, while still summing over all items.^24^ Alternatively, a population model of the number line, could obtain U-shape weights as a result of lateral inhibition between units that respond to similar values. Finally, one can modify the mid-range strategy by making it probabilistic, by including a probability parameter to fail in detecting the maximum/ minimum in the sequence and then recursively select the value next in rank. Future studies with larger number of participants per group, will be necessary to contrast such estimation mechanisms for the extraction of summary statistics of rapid numerical sequences and to characterize their individual differences. Below we discuss the relative efficiency of these two types of strategies, their relation with the two-pathway theory of numerical processing and the implications for the general field of decision-making.

### Estimation Efficiency of Numerical Averaging and Its Computation

Work in numerical cognition, has highlighted the presence of two processes or pathways in number processing: (i) exact vs. (ii) approximate (Dehaene and Cohen, 1991; Dehaene, 1992). While the former is based on symbolic representation and on rule based operations (Ashcraft, 1992), the latter is based on analog/magnitude representations and on perceptual type operations (Dehaene et al., 1998; Dehaene, 2007; Piazza et al., 2007). For example, both numerical symbols and numerosity (dot) displays activate the same distance-dependent approximate number representation in the parietal cortex (Piazza et al., 2007). Moreover, an analog representation of symbolic numbers is also supported by distance-effects in the comparison of symbolic numbers (Moyer and Landauer, 1967; Dehaene et al., 1990) and by data from patients with dyscalculia (Dehaene and Cohen, 1991). Finally, it was suggested that this analog representation of magnitudes is part of a core system for numerical processing, which is shared with other animal species (and non-verbal humans) and grounds the processing of symbolic representations (Feigenson et al., 2004; see also Verguts and Fias, 2004 for a computational modeling illustration).

While most previous work has focused on mathematical operations, such as addition or subtraction (Barth et al., 2008), here we have focused on averaging [see Katzin et al., 2021, for a study showing averaging of numerosity (dot) displays]. Obviously, one way (a symbolic one) to compute the numerical average of a sequence of numbers is via summation and division by *n*. The numerical cognition literature discussed above, suggests an alternative type of estimation: a population averaging of the analog magnitude representation, as was shown to be possible for perceptual properties (Ariely, 2001; Dakin, 2001; Parkes et al., 2001; Chong and Treisman, 2005; Haberman and Whitney, 2011). Thus, the approximate and the exact pathways may map into the types of models we have contrasted here, with the exact pathway mapping on to the (rule-based) mid-range strategy, and the approximate pathway onto the holistic estimation of all (but noisy) samples. A central question that can be raised is which of these pathways or processes is more efficient for tasks that require the extraction of summary statistics from rapid sequences. Note that this is not straightforward, as the two processes face a tradeoff. While the holistic model takes all items into account, it is subject to significant encoding noise on each item. By contrast, the mid-range strategy, relies on only two items, but due to its symbolic nature it is subject to minimal encoding noise. Motivated by this tradeoff, we have examined the data for association (across participants) between task precision and strategy use. We found that participants who were classified to the holistic model, had a higher precision in the task (mean RMSD = 7.2 and 8.4 for subjects classified as holistic and as mid-range, respectively). ^25^

### Implications for Decision-Making

The obvious importance of having a mechanism that automatically extracts summary statistics of rapid sequences of numerical payoffs, is that one can rely on it for preference formation, in situations that do not require explicit estimations. Indeed, statistical summaries are central to normative preference, as formalized by the prominent risk-return model, according to which the attractiveness of a risky alternative is an additive function of the alternative's average reward and its risk—that is, between the mean and the variance of the payoff distribution (Markowitz, 1952; Weber, 2010). While most frequently, preferences are probed via choices between sets of alternatives, in which the canonical models involve some type of accumulation over sampled valued (Townsend and Busemeyer, 1993; Roe et al., 2001; Usher and McClelland, 2004; Stewart and Simpson, 2008; Krajbich et al., 2012; Bhatia, 2013), it is also possible to probe relative preferences for individual alternatives using an analog (like) rating scale (Krajbich et al., 2012; see also Becker et al., 1964 for the BDM procedure).

Moreover, a number of studies have already indicated that summary statistics are used by participants to automatically form preferences for alternatives that are associated with rapid sequences of values. For example, in a pioneering study, Betsch et al. (2001) have shown that when exposed to information on the returns of several stocks, which are presented as distractors (and the task does not require to attend or rely on them^26^), the participants form automatic preferences that reflect their summary statistics. In a follow up study, Brusovansky et al. (2018), have shown that the preferences in tasks similar to the one we used here, are best reflected by the sequence-average. Finally, Vanunu et al. (2019), have extended this to risk preferences, by showing that preferences for rapid sequences of values presented one at a time, are well-accounted by a risk-return model, in which the preference is a weighted average of the average and the standard-deviation of the value estimate. We thus propose that such summary statistics can be holistically computed in an automatic way when we are exposed to alternatives consisting of numerical sequences (see also Betsch et al., 2001; Betsch and Glöckner, 2010; Brusovansky et al., 2018; Vanunu et al., 2019), contributing a general preference toward alternatives associated with high averages (and low variances).

Finally, we suggest that the automatic extraction of summary statistics of sets of values (payoffs or affective attributes) can provide a mechanism for intuitive decisions or forecasts, in which participants appear to rely on “gut-feeling” resulting in decision outcomes that sometimes exceed those of conscious deliberations (Bechara and Damasio, 2005; Dijksterhuis and Nordgren, 2006; but see Wilson and Schooler, 1991; González-Vallejo et al., 2008; Lee et al., 2009; Usher et al., 2011; Pham et al., 2012; Rusou et al., 2013).

## Data Availability Statement

The original contributions presented in the study are included in the article/Supplementary Material, further inquiries can be directed to the corresponding author/s.

## Ethics Statement

The studies involving human participants were reviewed and approved by Ethics Committee of Tel Aviv University. The patients/participants provided their written informed consent to participate in this study.

## Author Contributions

DR and MU conceived of the presented idea. DR and MG performed the analysis and the computational modeling. All authors discussed the results and contributed to the final manuscript.

## Funding

This work was supported by Israel Science Foundation 1413/17.

## Conflict of Interest

The authors declare that the research was conducted in the absence of any commercial or financial relationships that could be construed as a potential conflict of interest.

## Publisher's Note

All claims expressed in this article are solely those of the authors and do not necessarily represent those of their affiliated organizations, or those of the publisher, the editors and the reviewers. Any product that may be evaluated in this article, or claim that may be made by its manufacturer, is not guaranteed or endorsed by the publisher.

## Footnotes

^1^As we discuss below, paying attention to outliers (in particular the max and the min of a set) is also an effective strategy to extract summary statistics (Myczek and Simons, 2008).

^2^As we only aim to examine sensitivity to relative-variance (i.e., to ordinal comparison of variance magnitudes) this is the same as relative-SD, relative-spread (or dispersion). We will use these terms interchangeably in the text (but see Methods for how this was presented to participants).

^3^We assume that subjects maintain 2-3 items in WM (Cowan, 2001) and are able to compute the average of 2-3 numbers with a reasonable precision.

^4^The term “analytic” refers to the fact that subject to WM-capacity limitation, one relies on a symbolic, rule-based and error less computation. However, since this strategy does not use all of the information available, one may also think of it as an intuitive one (Kruglanski and Gigerenzer, 2011).

^5^We may equally label this as a min-max heuristic, as it estimates the average based on the min and the max of each sequence. As this is the same as computing the *mid-range* (a term used in the heuristic literature) we will keep this label.

^6^The Pearson correlation between actual and estimated values, and the RMSD (root mean square deviations) provide complementary but well correlated aspects of precision. The RMSD (but not the correlation) is affected by systematic biases, while correlation (but less the RMSD) is affected by the range of the values. Here we report both, but we focus on RMSD when examining differences in precision with sequence-length (as this variable affects the range).

^7^According to this heuristic (based on the min and the max of each sequence) only these two items (min and max) should have high weights in the estimation.

^8^Skewed distributions of values increase the gap between the average and the mid-range making the mid-range strategy a poorer estimator of the average.

^9^This N value was larger than the maximum used in all the experiments reported in these articles. Note that in study-1 of Pleskac et al. (2019) N=40 participants were tested. However, these participants were randomly allocated to two conditions, resulting in 20 participants per condition. With regards to averaging our experiments can also be thought of as conditions (and thus we have a total N of 100, with 25 participants per condition).

^10^As shown in Figure 6A, the mean of the actual sequences varies continuously in the range (30-70).

^11^We also estimate the U-shape pattern by extracting a curvature parameter for the parabolic fit of the ranked regression, for each participant. We found a very high correlation (*r*=.93, *p*<.001) between the curvature parameter and the difference between the mean of outlying and inlying ranks. See Supplementary Figure 10.

^12^We intentionally used the term “spread” (rather than variance or SD) in the instructions, appealing to the subjects' pre-experimental knowledge in order to prevent participants from engaging in an explicit calculations and to preclude dependence on math skills.

^13^Note that despite the invariance of the encoding noise to sequence-length the estimation-precision improves with sequence-length due to noise-averaging (see Figure 2).

^14^The (a) intercept can correspond to subjective biases in the mapping of the internal estimation to the external scale, while the (b) slope to a ‘regression to the mean’ that is normative when the distribution of sequences is centered around a mid-range value (50 in our case). A value of *b*=*1* corresponds to ***no*** regression to the mean, while a value of *b*=*0*, would correspond to ***full*** regression to the mean, in which the actual values of the sequences are not weighted at all.

^15^The encoding noise corresponds to the encoding of the symbolic number into an analog magnitude response (Dehaene, 2007).

^16^Note that the responses are provided via the mouse on a continuous scale.

^17^Note that for this comparison we grouped Experiments 1A and B together and Experiments 2 and 3 together, resulting in sample size of N=50 per group.

^18^The observed power of our main results is quite high. For example, the observed power of the classification of Experiments 1a and b (complex models) being different than chance is 1 (two-sided binomial test against.5, α =.05) and of the comparison between the classification of Experiments 1a and b and Experiment 2 and 3 (complex models) is.99 (difference between proportions, α =.05).

^19^The comparison between Experiment 1A and 1 B (which concerns the shape of the value distribution) needs to be qualified by the smaller number of participants (N=25, per group).

^20^Note that by grouping this analysis over Experiments 2 and 3, we are considering a sample of N=50 participants.

^21^This analysis was done using the data of experiments 1a, 2 and 3. We did not use experiment 1b due to the fact that the performance (precision) in this experiment was much lower than in the other experiments and this could introduce artifacts into the correlation results. We also excluded 12 participants that had a negative outliers-inliers score which indicates relying on the inliers samples more than the outliers (i.e., robust averaging; De Gardelle and Summerfield, 2011). This is a third strategy which perhaps should be addressed in future studies but is beyond our focus here (precision as a function of mid-range compared to normative holistic strategies).

^22^In this study we were also limited by COVID contingencies in the number of participants we were able to recruit to our experiments.

^23^Glöckner and Betsch (2008) label such strategies as ‘automatic’, while we label them as ‘holistic’. We believe those terms involve similar concepts, as the both rely on a normative estimation (that weights across all items, in parallel, rather than sequentially) and free from WM-capacity limitations.

^24^For example, we have explored a model in which the numbers are weighed with Beta functions over the interval 0-100.

^25^In principle a subject classified as Mid-range, can have better accuracy than one classified as Holistic, if the latter, resorts to a higher encoding noise. The results reported here indicate that this is not the case.

^26^To do this, the study relies on a 1-trial per subject methodology (since after 1 trial takes place the subjects become aware that the values of the sticks are task relevant).
