# Supplementary material for: Extracting Summary Statistics of Rapid Numerical Sequences
Source: Front Psychol. 2021 Oct 1;12:693575. doi: 10.3389/fpsyg.2021.693575 (PMC8517333; doi:10.3389/fpsyg.2021.693575)
Supplement: Supplementary file 1 [file Data_Sheet_1.pdf]

Supplementary Information

Model Comparison

Model Fitting

Optimization procedure. The free parameters of the normative-holistic and the mid-range models were fitted to the data of each participant separately, using maximum likelihood estimation. We first constructed an n-dimensional grid (n is the number of free parameters for each model), with a ranging from -20 to 20 in increments of 10, b ranging from 0 to 3 in increments of .75, σ_e (only for the normative-holistic model) and σ_m ranging from 0 to 10 with increments of 2.5, σ_v (complex normative-holistic model) and σ_c (complex mid-range model) ranging from 0 to 1 with increments of 0.1. This grid was searched exhaustively, and for each set of parameters, θ_j, the likelihood was calculated based on a Gaussian probability distribution function:

$$L(\theta_j) = \prod_{i=1}^{N} \frac{1}{\sigma\sqrt{2\pi}} e^{\frac{-1}{2}\left(\frac{x_i-\mu_i}{\sigma}\right)^2}$$

where N is the number of trials, x_i is the subject's estimated average in each trial, μ_i is the predicted average by the model excluding noise, and σ is the standard deviation such that $\sigma^2 = \sigma_e^2 + \sigma_m^2$ for the normative-holistic model (see equation 3) and $\sigma^2 = \sigma_m^2$ for the Mid-range model. The five parameters sets that had the highest likelihood were fed as starting points to a Simplex minimization routine, in which the cost function was defined as the negative log-likelihood. The mean best-fitting parameters (averaged across participants) are shown in Table S3.

Model selection. In order to evaluate the quantitative fits of the models, we used two methods: i) Akaike Information Criterion (AIC; Akaike, 1974), and ii) Bayesian Information Criterion (BIC; Schwarz, 1978, Raftery, 1995), these selection criteria implement a trade-off between model goodness of fit and complexity by penalizing additional free parameters according to the following formulas:

$$AIC = -2 \cdot LL + 2 \cdot k$$

$$BIC = -2 \cdot LL + k \cdot log(N)$$

where LL is the log-likelihood for the best fitting parameters, k is the number of free parameters and N is the number of trials. AIC/BIC differences exceeding 10 are

considered decisive evidence in favor of the model with the lower numerical values (Burnham & Anderson, 2002; Raftery 1995). To calculate the AIC/BIC measures at the group level, we used the above formulas, but with the group *LL*, *k*, and *N*, which were obtained by summing the individual values across the group.

Data vs. models

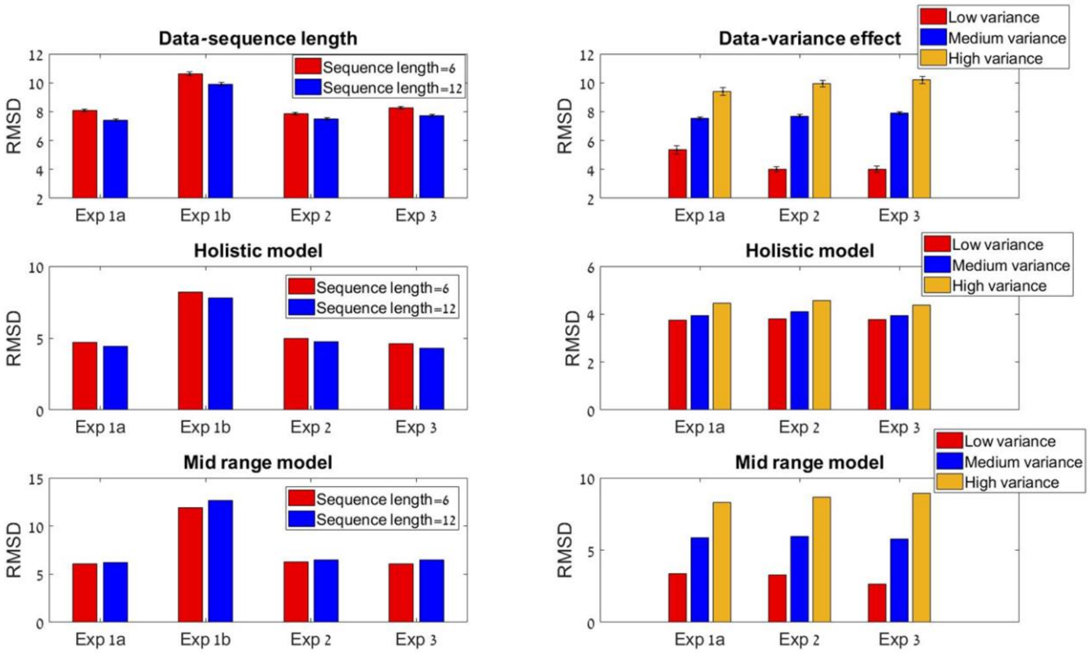

Figure S1. *Simulation using the average values of each parameter across participants. TOP-row: data, Midle-row: Holistic model; Bottom-row: mid-range model; Sequence-length: left column; Sequence-variance: right column.*

Experiment 1a

Table S1. *Comparison between the* ***AIC*** *and* ***BIC*** *values of the Normative-holistic and Mid-range models in* ***Exp. 1a****. The values are rounded to the nearest integer.*

Subject	Normative-holistic model AIC	Normative-holistic model BIC	Mid-range model AIC	Mid-range model BIC
1	**1932**	**1947**	2077	2088
2	**1998**	**2013**	2025	2036
3	1967	1982	**1925**	**1936**
4	**1657**	**1672**	1765	1776
5	**2165**	**2180**	2176	2187
6	**2216**	**2231**	2251	2262
7	**2025**	**2040**	2069	2080
8	**1937**	**1952**	1963	1974
9	**2230**	**2245**	2237	2248
10	**1781**	**1796**	1847	1858
11	**2207**	**2222**	2220	2231
12	**1850**	**1865**	1925	1936
13	**1912**	**1927**	1957	1968
14	**1884**	**1899**	1923	1934
15	**2147**	**2162**	2168	2180
16	**2031**	2046	2032	**2043**
17	**2050**	**2065**	2121	2132
18	**1862**	**1877**	1908	1919
19	**1951**	**1966**	1968	1979
20	**1929**	**1944**	1944	1955
21	**1980**	**1995**	1997	2008
22	**1947**	**1962**	2040	2051
23	**1831**	**1846**	1851	1862
24	**1911**	**1926**	1942	1954
25	**2098**	**2112**	2166	2177

Experiment 1b

Table S2. *Comparison between the **AIC** and **BIC** values of the Normative-holistic and Mid-range models in **Exp. 1b**. The values are rounded to the nearest integer.*

	Normative-holistic model		Mid-range model	
Subject	AIC	BIC	AIC	BIC
1	**2311**	**2326**	2430	2441
2	**2091**	**2106**	2151	2162
3	**1861**	**1876**	2079	2090
4	**2181**	**2195**	2257	2268
5	**2153**	**2168**	2221	2232
6	**2097**	**2112**	2242	2253
7	**2223**	**2238**	2286	2297
8	**2227**	**2241**	2252	2263
9	**2281**	**2296**	2331	2342
10	**1991**	**2006**	2118	2130
11	**2051**	**2066**	2228	2239
12	**2059**	**2074**	2191	2202
13	**2311**	**2325**	2353	2364
14	**2366**	**2381**	2385	2396
15	**2246**	**2261**	2274	2285
16	**2277**	**2292**	2364	2375
17	**2446**	**2460**	2472	2484
18	**2035**	**2049**	2109	2120
19	**1956**	**1971**	2008	2020
20	**2198**	**2213**	2294	2305
21	**2036**	**2051**	2115	2126
22	**1854**	**1869**	2123	2134
23	**1829**	**1844**	1886	1897
24	**2222**	**2237**	2362	2373
25	**2251**	**2265**	2351	2362

Experiment 2

Table S3. *Comparison between the **AIC** and **BIC** values of the Normative-holistic and Mid-range models in **Exp. 2**. The values are rounded to the nearest integer.*

	Normative-holistic model		Mid-range model	
Subject	AIC	BIC	AIC	BIC
1	1877	1892	**1856**	**1867**
2	**1957**	**1972**	1997	2008
3	**2010**	**2025**	2042	2054
4	**1881**	**1896**	1901	1912
5	2021	2036	**1993**	**2004**
6	2078	2093	**2061**	**2072**
7	**2068**	**2082**	2104	2116
8	**1868**	**1883**	1893	1904
9	1882	1896	**1869**	**1880**
10	**1882**	**1896**	1996	2007
11	**1892**	**1906**	1918	1929
12	**1951**	**1966**	1980	1992
13	1963	1977	**1953**	**1965**
14	**1909**	**1924**	1988	1999
15	2054	2069	2054	**2065**
16	**1860**	**1875**	1879	1890
17	**1926**	**1940**	1986	1997
18	2031	2046	**2004**	**2015**
19	2040	2055	**2028**	**2039**
20	**1957**	**1972**	1996	2007
21	**1881**	**1895**	1886	1897
22	**2193**	**2208**	2208	2219
23	**1953**	**1968**	1979	1991
24	2051	2066	**2041**	**2052**
25	**2014**	**2029**	2019	2030

Experiment 3

Table S4. *Comparison between the* ***AIC*** *and* ***BIC*** *values of the Normative-holistic and Mid-range models in* ***Exp. 3****. The values are rounded to the nearest integer.*

	Normative-holistic model		Mid-range model	
Subject	AIC	BIC	AIC	BIC
1	**1931**	**1945**	1946	1957
2	**1924**	**1939**	1963	1974
3	**1872**	**1887**	1917	1928
4	**1968**	**1982**	2015	2027
5	**1983**	**1998**	2009	2020
6	**1967**	**1982**	2044	2055
7	**2270**	**2285**	2294	2305
8	**1891**	**1906**	1898	1909
9	2028	2043	**1946**	**1957**
10	2031	2046	**2002**	**2013**
11	1950	1965	**1918**	**1929**
12	1943	1958	**1920**	**1931**
13	**2140**	**2155**	2148	2159
14	2049	2064	2049	**2060**
15	**2251**	**2266**	2265	2276
16	**1899**	**1914**	1933	1944
17	**2258**	**2273**	2286	2297
18	**1835**	**1849**	1858	1869
19	**2121**	**2135**	2174	2185
20	**2189**	**2204**	2197	2208
21	**1991**	**2006**	2009	2020
22	**1800**	**1815**	1883	1894
23	**2128**	**2143**	2149	2160
24	**1830**	**1845**	1887	1898
25	**1905**	**1920**	1933	1943

Experiment 1a parameters histogram for holistic model

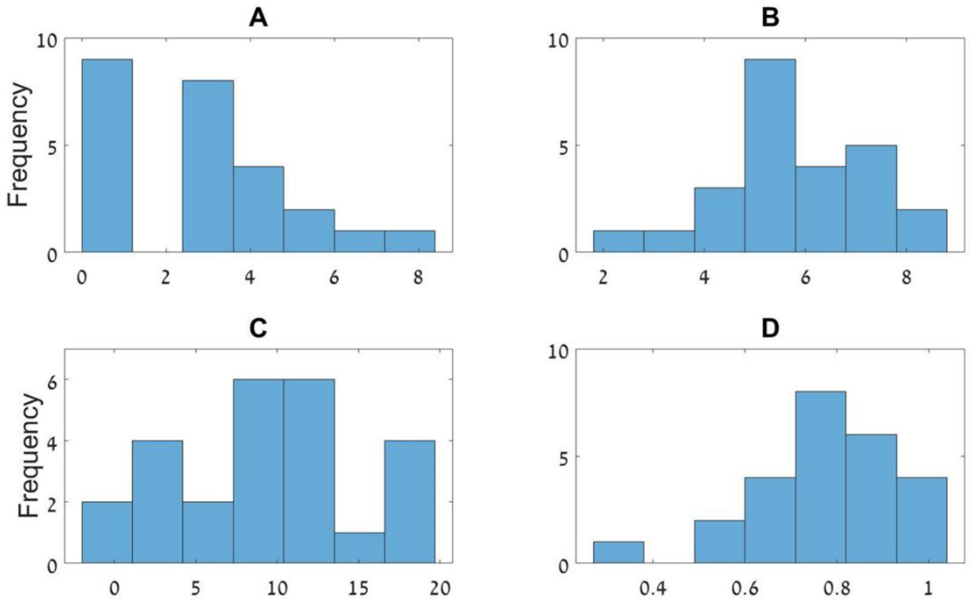

Figure S2. *Parameters histogram. A) values distribution of the encoding noise parameter. B) values distribution of the motor noise parameter. C) values distribution of the intercept parameter. D) values distribution of the slope parameter.*

Experiment 1a parameters histogram for Mid-range model

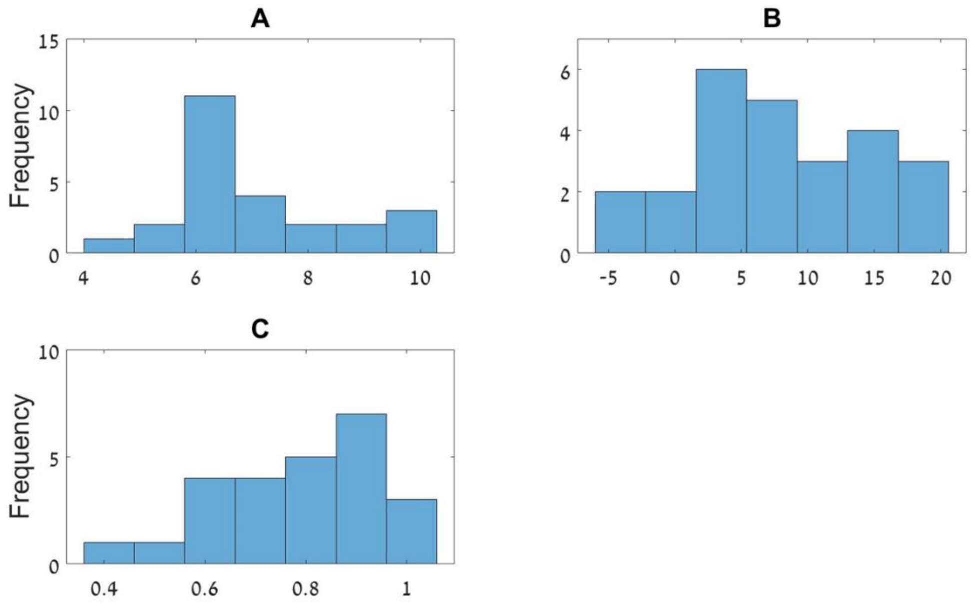

Figure S3. *Parameters histogram. A) values distribution of the motor noise parameter. B) values distribution the intercept parameter. C) values distribution of the slope parameter.*

Experiment 1b parameters histogram for holistic model

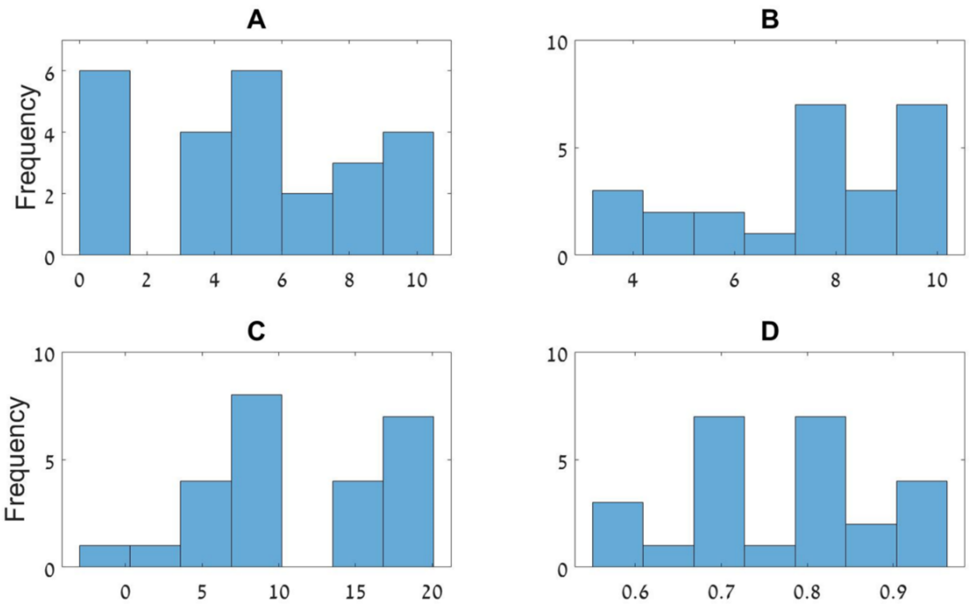

__Figure S4.__ Parameters histogram. A) values distribution of the encoding noise parameter. B) values distribution of the motor noise parameter. C) values distribution of the intercept parameter. D) values distribution of the slope parameter.

Experiment 1b parameters histogram for Mid-range model

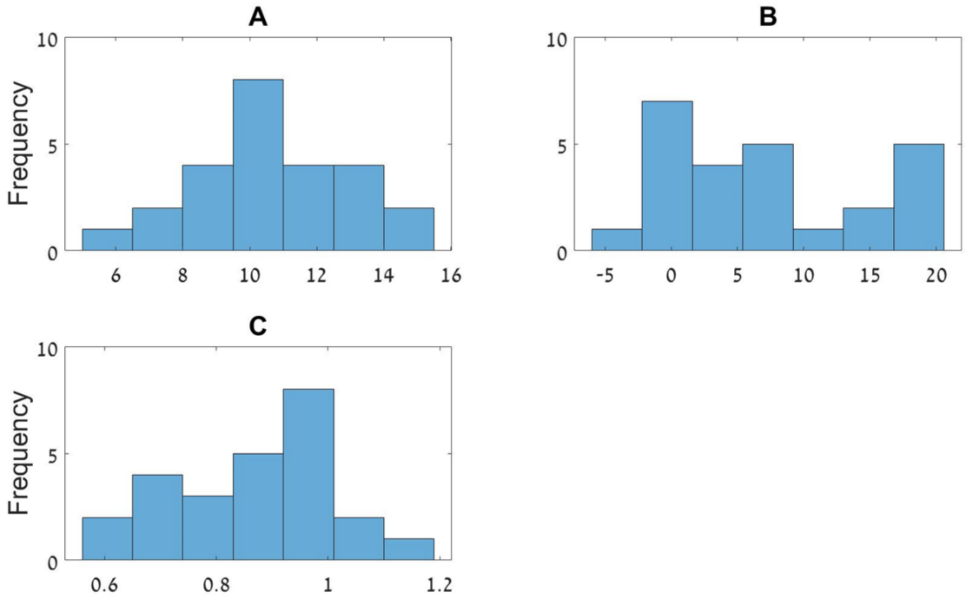

__Figure S5.__ Parameters histogram. A) values distribution of the motor noise parameter. B)

values distribution of the intercept parameter. C) values distribution of the slope parameter.

Experiment 2 parameters histogram for holistic model

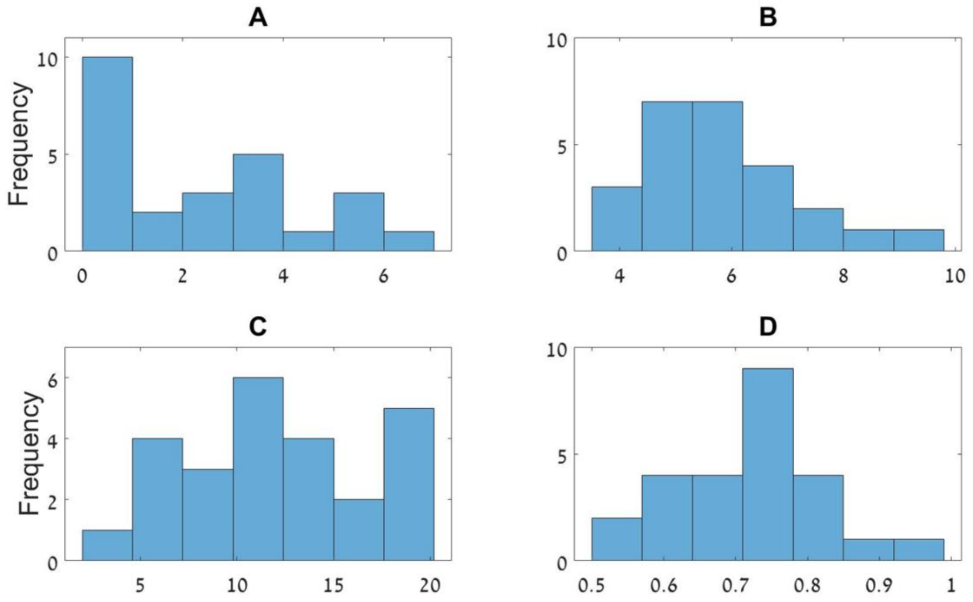

Figure S6. *Parameters histogram. A) values distribution of the encoding noise parameter. B) values distribution of the motor noise parameter. C) values distribution of the intercept parameter. D) values distribution of the slope parameter.*

Experiment 2 parameters histogram for Mid-range model

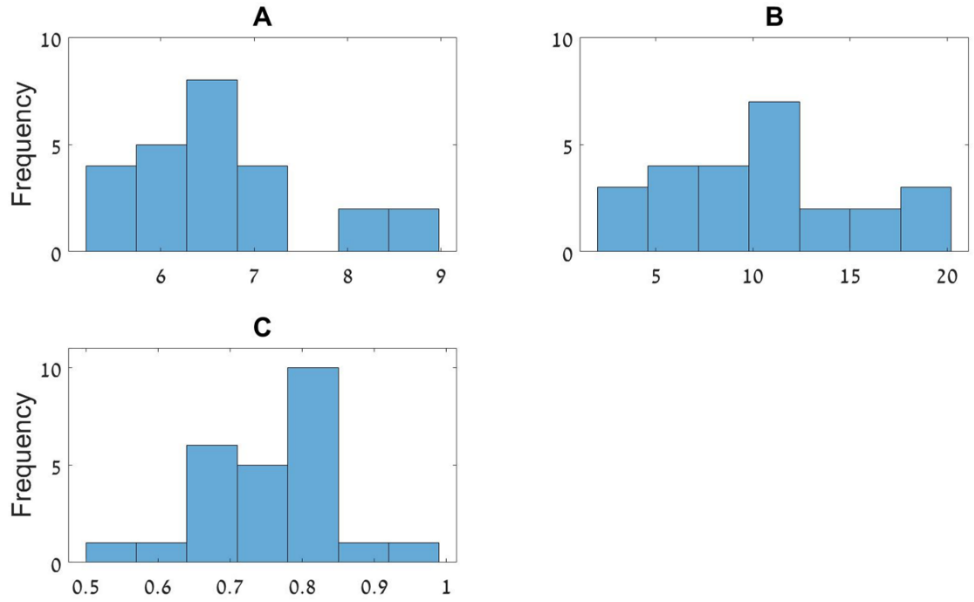

Figure S7. *Parameters histogram. A) values distribution of the motor noise parameter. B) values distribution of the intercept parameter. C) values distribution of the slope parameter.*

Experiment 3 parameters histogram for holistic model

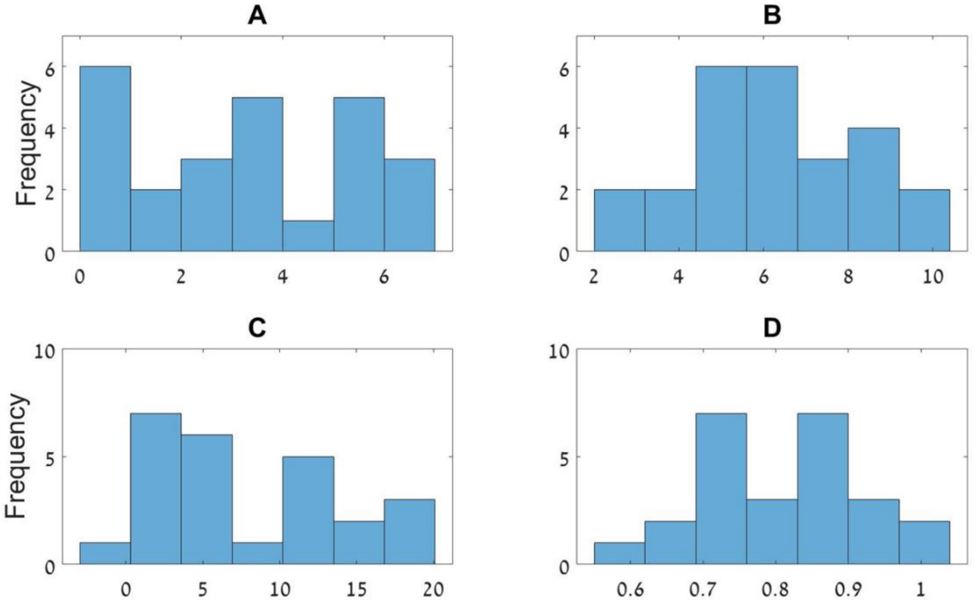

Figure S8. *Parameters histogram. A) values distribution of the encoding noise parameter. B) values distribution of the motor noise parameter. C) values distribution of the intercept parameter. D) values*

distribution of the slope parameter.

Experiment 3 parameters histogram for Mid-range model

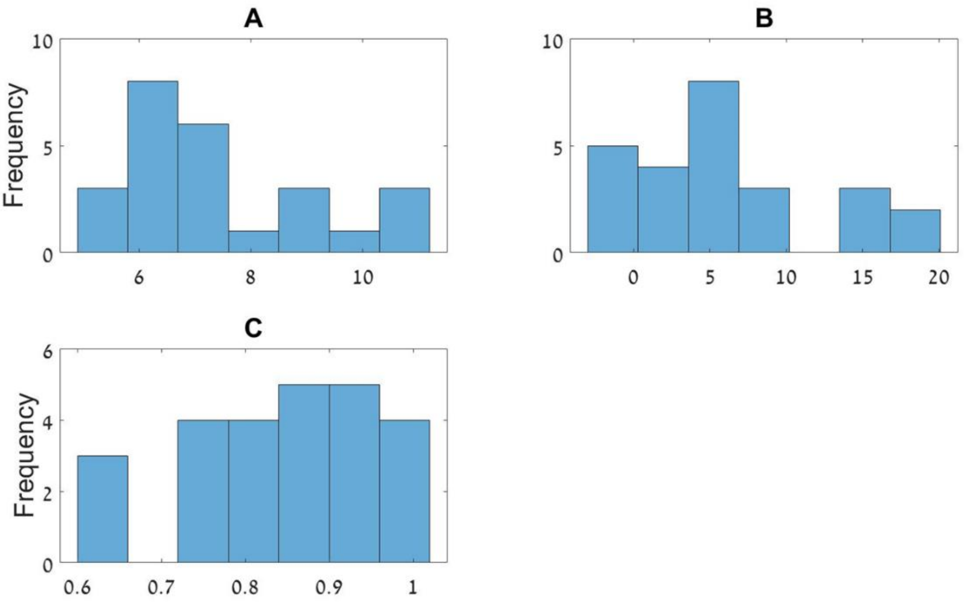

Figure S9. *Parameters histogram. A) values distribution of the motor noise parameter. B) values distribution of the intercept parameter. C) values distribution of the slope parameter.*

Classification recovery

In order to probe the classification recovery of the models we first created synthetic data using the holistic model or the mid-range model (30 different "subjects" for each model). We then fitted the synthetic data to each model using the same fitting procedure we used for the real data and compared the BIC measure of the two models. In 98% of the times the model that had a better BIC fit was the model we used to create the data. In order to generate the data for both models we constructed an n-dimensional grid (n is the number of free parameters for each model), with a ranging from -20 to 20 in increments of 1.33, b ranging from 0 to 3 in increments of .1, σ_e (only for the normative-holistic model) and σ_m ranging from 0 to 10 with increments of 0.5. We randomly sampled a point from the grid for each synthetic subject.

U-shape patterns measures

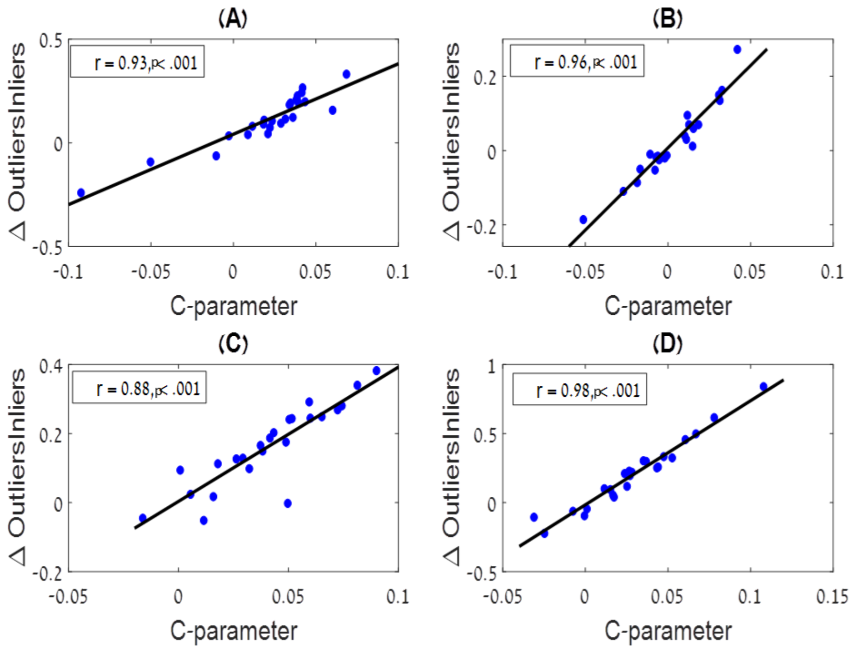

Figure S10. *Correlations between curvature parameter for the parabolic fit of the ranked regression and the difference between the mean of outlying and inlying ranks. The high correlations indicate that both measures are similar in estimating the U-shape patterns. A-D represents experiments 1a, 1b, 2 and 3 respectively.*
